# Advances in the study of emodin: an update on pharmacological properties and mechanistic basis

**DOI:** 10.1186/s13020-021-00509-z

**Published:** 2021-10-10

**Authors:** Qi Zheng, Shuo Li, Xiaojiaoyang Li, Runping Liu

**Affiliations:** 1 School of Chinese Materia Medica, Beijing University of Chinese Medicine, 11 Bei San Huan Dong Lu, Beijing, 100029 China; 2 School of Life Sciences, Beijing University of Chinese Medicine, 11 Bei San Huan Dong Lu, Beijing, 100029 China

**Keywords:** Emodin, Pharmacology, Toxicology, Pharmacokinetics, Modification

## Abstract

*Rhei Radix et Rhizoma,* also known as rhubarb or *Da Huang*, has been widely used as a spice and as traditional herbal medicine for centuries, and is currently marketed in China as the principal herbs in various prescriptions, such as *Da-Huang-Zhe-Chong* pills and *Da-Huang-Qing-Wei* pills. Emodin, a major bioactive anthraquinone derivative extracted from rhubarb, represents multiple health benefits in the treatment of a host of diseases, such as immune-inflammatory abnormality, tumor progression, bacterial or viral infections, and metabolic syndrome. Emerging evidence has made great strides in clarifying the multi-targeting therapeutic mechanisms underlying the efficacious therapeutic potential of emodin, including anti-inflammatory, immunomodulatory, anti-fibrosis, anti-tumor, anti-viral, anti-bacterial, and anti-diabetic properties. This comprehensive review aims to provide an updated summary of recent developments on these pharmacological efficacies and molecular mechanisms of emodin, with a focus on the underlying molecular targets and signaling networks. We also reviewed recent attempts to improve the pharmacokinetic properties and biological activities of emodin by structural modification and novel material-based targeted delivery. In conclusion, emodin still has great potential to become promising therapeutic options to immune and inflammation abnormality, organ fibrosis, common malignancy, pathogenic bacteria or virus infections, and endocrine disease or disorder. Scientifically addressing concerns regarding the poor bioavailability and vague molecular targets would significantly contribute to the widespread acceptance of rhubarb not only as a dietary supplement in food flavorings and colorings but also as a health-promoting TCM in the coming years.

## Introduction

*Rhei Radix et Rhizoma* (synonym: rhubarb, Chinese name: *Da Huang*) is the dry root and rhizome of three official *Rheum species*: *R. palmatum* L., *R. tanguticum* Maxim. ex Balf. and *R. officinale* Baill.. Rhubarb is mainly produced in the high-altitude mountainous area of temperate and subtropical Asia, including but not limited to the northwest and southwest of China (Gansu province, Qinghai province, Sichuan province, and Tibet Autonomous Region). Historically, after first traveled to Europe in the early seventeenth centuries, culinary or garden rhubarb, the stalk of *Rheum rhaponticum* L. (synonym: *R.hybridum*, *R.X cultorum*), is widely cultivated in North America, Northern Europe as well as Oceania, and gains popularity on account of its high nutritional and pharmaceutical value [1, 2]. Rhubarb was first recorded in *Shen Nong Ben Cao Jing*, the earliest systematic monography concerning Traditional Chinese Medicine (TCM), and has been clinically practiced for over the past 2000 years due to its pharmacological effects against constipation, inflammation, virus infection, diabetes, and tumor [3–5]. In addition, rhubarb has also been concomitantly used with other herbal medicines in traditional Chinese prescriptions to improve therapeutic effects or reduce side effects. Clinically, several widely prescribed Chinese patent medicines and prescriptions are made from or containing rhubarb, including *Yin-Chen-Hao* decoction, *Da-Cheng-Qi* decoction, *Da-Huang-Zhe-Chong* pills, *Niu-Huang-Jie-Du* tablets, and *Liu-Wei-An-Xiao* capsules [6–8].

Numerous bioactive ingredients and plant secondary metabolites have been isolated and identified in rhubarb, including anthraquinones, anthrones, stilbenes, butyrophenones and chromones, flavonoids, tannins, polysaccharides, volatile oils, and other compounds. Among them, anthraquinones are the major characteristics and abundant pharmacodynamic components isolated from rhubarb, which can be further separated into free anthraquinones, such as rhein, emodin, chrysophanol, aloe-emodin and physcion, and bound anthraquinones [9]. Since merging studies have demonstrated that emodin (1,3,8-trihydroxy-6-methylanthraquinone, C_15_H_10_O_5_) exhibited significant pharmacological functions and effective clinical curative effects, it has long been considered as the principal pharmacodynamic component of rhubarb. Viewed from its botanical origin, the content of emodin in *R. palmatum* L. is the highest among the three sources of rhubarb [10]. Additionally, it is also noteworthy that emodin has been identified in 17 families of natural plants. In addition to the above-mentioned Polygonaceae (*R. palmatum* L., *R. tanguticum* Maxim. ex Balf., *R. officinale* Baill.), emodin was isolated from Polygonaceae (*Polygonum cuspidatum* Sieb.et Zucc., *Polygonum multiflorum* Thunb.) [11, 12], Fabaceae (*Cassia obtusifolia* L., *Cassia tora* L.) [13] and Rhamnaceae (*Rhamnus* spp.) [14]. Emodin is considered to possess a wide variety of pharmacological benefits, including anti-cardiovascular, anti-cancer, anti-diabetic, and anti-fibrosis effects, and was clinically used for the treatment of bronchial asthma, encephalitis, nasopharyngeal carcinoma (NPC), and diabetic cataract [15–19]. In the meantime, an increasing number of toxicologic studies reported that abuse of emodin tends to initiate adverse effects like hepatotoxicity and nephrotoxicity [20, 21]. The cellular and molecular mechanisms of emodin-mediated organ toxicity remain an unclear territory that warrants further exploration.

In the present review, we summarize the current state of experimental evidence for the pharmacological and toxic effects of emodin, mainly focusing on those studies exploring underlying mechanisms and molecular targets. We also highlight recent advances in the study of structure–activity relationships, structural modification of emodin and modern delivery approaches aiming to improve the targeting and pharmacokinetic properties of emodin. This review aims to point out the shortcomings of previous studies and to offer intriguing insights into the discovery of emodin-based novel drugs which have a promising prospect in clinical application.

## Pharmacological effects of emodin

### Anti-oxidant effects

Emodin shows striking O_2_^•−^ and ^•^NO capture capacity and thus works as a powerful antioxidant for neuroprotection purposes and for treating inflammatory disorders, which is well-characterized by numerous studies and has been well-summarized by previous reviews. Several recent studies further shed novel light on the underlying mechanisms. By employing a range of new technologies including X-ray crystallography, density functional theory, cyclic voltammetry, and rotating ring-disk electrode method, Rossi et al*.* clarified that expect for forming a stable complex after interacting with the hydroxyl at position 3, the π–π interaction of superoxide and quinone moiety also helped to transfer one electron to the ring and induced emodin aromatization and oxidation, thus exhibited strong free radical scavenging and antioxidant activities [22].

### Anti-inflammatory effects

**Fig. 1:**
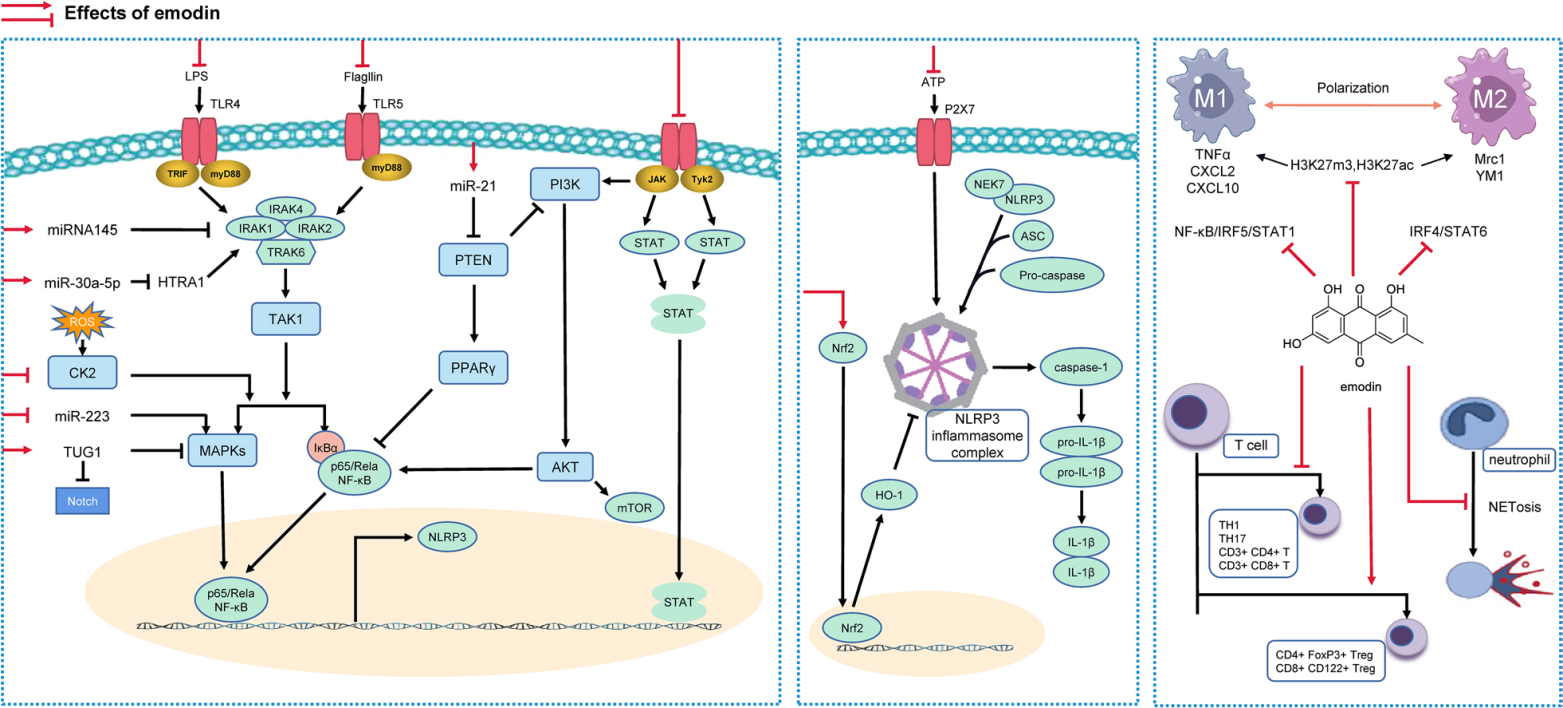
Networks of molecular signaling underlying anti-inflammatory and immunoregulative effects of emodin. Emodin limits immune and inflammation abnormality through inhibition of pro-inflammatory signaling pathways (such as TLR/NF-κB, PI3K/AKT, and JAK/STAT3) and NLRP3 inflammasome activation, induction of apoptosis, and regulation of microRNA expression as well as immune cells differentiation

Inflammation is a comprehensive array of pathophysiological responses to stressful stimuli, which may lead to local tissues or even systemic injuries when it is excessive and uncontrolled. There was a growing number of evidence showing that emodin exerted protective effects against tissue or organ damage through inhibiting exaggerated inflammatory responses (Fig. 1). Nuclear factor kappa-B (NF-κB) is considered as the master regulator of inflammatory response and serves crucial roles in transcriptionally activating a bunch of cytokines, chemokines, pro-inflammatory effectors as well as inflammatory cells. It can be activated by toll-like receptor (TLR) signal through myeloid differentiation factor88 (MyD88)-dependent pathway, including the degradation of IkBα, a cytoplasmic inhibitor of NF-κB, and sequentially NF-κB p65 nuclear translocation [23]. Emodin (10 μM) was found to inhibit the calcific transformation of human aortic valve interstitial cells and interfere with the calcification events during acute inflammatory responses under the induction of tumor necrosis factor-alpha (TNF-α). RNA-sequencing and GO functional annotations revealed that emodin significantly suppressed NF-κB pathway and down-regulated bone morphogenetic protein 2 expression by inhibiting TNF, tumor necrosis factor receptor-associated factors, and p65 protein synthesis [24]. Luo et al*.* firstly found that emodin reduced the expression of TLR5 and MyD88, up-regulated IκB levels, as well as inhibited the nuclear translocation of NF-κB p65 to ameliorate ulcerative colitis symptoms, via suppressing flagellin-TLR5-NF-κB signaling pathway [25]. Emodin (5–20 mg/kg) also was reported to markedly inhibit inflammatory response and subsequent liver injury induced by lipopolysaccharide (LPS), via suppressing TLR4-mediated MyD88-TRIF signaling pathways and reducing the expression of TNF-α and interleukin (IL)-6 [26]. Moreover, emodin (25 μM) markedly suppressed the LPS-triggered up-regulation of intercellular cell adhesion molecule-1 (ICAM-1), monocyte chemoattractant protein-1 (MCP-1) and TNF-α, and reversed the down-regulation of PPARγ in activated RAW264.7 cells, suggesting the implication of PPARγ pathway in emodin-mediated inactivation of NF-κB [27]. In primary uterine and endometrium epithelial cells isolated from leptospira-infected mice, emodin (40 μg/ml) inhibited the expression of TNF-α, IL-1β, and IL-6 and suppressed the phosphorylation of p38, p65, ERK and JNK via regulating NF-κB and MAPK signaling pathways [28]. Analogously, emodin (2 or 5 mg/kg) exerted renoprotective effects in ischemia reperfusion injured mice through limiting the activation of protein kinase 2 (CK2), a pro-inflammatory regulator in NF-κB and MAPK pathways [29].

In the past decade, there has been a growing emphasis on the putative role of long-noncoding RNAs (lncRNAs) and MicroRNAs (miRNAs or miR), which are increasingly seen as promising transcriptional mediators related to gene expression and cell metabolism, in the progression and management of multiple inflammation-related pathophysiological states [30]. Taurine raised gene 1 (TUG1), a 7.1-kb lncRNA transcribed from chromosome 22q12, was identified to attenuate cold-induced liver injury by repressing apoptosis and inflammation [31]. Based on RNA-sequencing and lncRNA microarray, Katsushima et al*.* revealed a reinforcing activation loop between Notch downstream factor MYC and lncRNA TUG1 for mutual stabilization of their gene expression, namely MYC functions as a transcriptional activator of TUG1 while TUG1 prevent MYC mRNA from degradation by quenching miR-145 [32]. Interestingly, emodin treatment (5–10 μM) was reported to induce TUG1 expression and inhibit the Notch and NF-κB signals and thus relieved LPS-induced inflammation in murine chondrogenic cell line (ATDC5). Additionally, the down-regulated expressions of Notch 1, Notch 2, Notch3, and the repressed phosphorylation of p65 and NF-κB induced by emodin were markedly reversed after TUG1 knockdown by transfection with sh-TUG1, which further consolidated the argument [33]. A most recent study in human fibroblasts cell line (WI-38) further demonstrated that emodin (5–20 μM) enhanced TUG1 expression and alleviated the LPS-evoked inflammatory damage via restraining the NF-κB and p38MAPK pathways [34].

MicroRNAs, a class of endogenous small non-protein-coding RNAs with approximately 19–23 nucleotides, function in post-transcriptional regulation of gene expression by binding to the 3’UTR of target mRNA, thus lead to mRNA degradation and translation inhibition at the same time [35, 36]. Serine protease high-temperature requirement A (HTRA) can repress transforming growth factor beta 1 (TGF-β1) protein level, which serves as a modulatory mechanism for the limitation of inflammatory responses [37]. Based on bioinformatic approaches, Xiang et al*.* elucidated that miRNA-30a-5p was an essential transcriptional brake to curb the expression of HTRA1 gene, concurrently repressing its protein translation. Interestingly, emodin (10–40 µM) significantly upregulated miRNA-30a expression in rat pancreatic acinar cells (AR42J) and further mitigated sodium taurocholate-induced inflammatory injury by downregulating HTRA1 [38]. Besides, IL-1 receptor associated kinase 1 (IRAK1) exerts critical roles in modulating innate immunity and inducing the expressions of inflammatory-related genes. A previous study has reported that emodin pre-treatment at 20 µM attenuated LPS-stimulated hepatic inflammatory injury via inducing miRNA-145-mediated translational suppression of IRAK1 and inhibiting following NF-κB signal in normal human liver cells (L-02) [39]. Moreover, miR-223 is regarded as a post-transcriptional regulator, which directly targeting a network of genes related to NF-κB pathway, including CACTIN, CARM-1, MCP-1, and UBE2G2, to promote innate immunity and inflammatory response [40]. Emodin treatment (15 μM) remarkably attenuated LPS-induced inflammatory lesion by down-regulating miR-223 expression and deactivating JNK signaling-related proteins p-Jnk and p-c-Jun in rat cardiomyoblast cells [41]. Emodin (20 μM) was also reported to reverse LPS-diminished miR-21 expression, thereby downregulated phosphatase and tensin homolog (PTEN) and blunted NF-κB signaling, and then alleviated LPS-induced human keratinocyte cells inflammation. Silencing of miR-21 significantly abrogated the inhibitory effects of emodin on inflammation, and further accelerated cell apoptosis and facilitated the expression of IL-1β and IL-6 [42].

Recognition of a wide range of pathogens challenges and cellular damages by inflammasomes leads to direct activation of caspase-1, and subsequent triggers the maturation of proinflammatory cytokines including IL-1β and IL-18 to engage in immune defense [43]. In addition to the above-mentioned mechanisms, it is noteworthy that emodin also alleviated inflammation by interfering with inflammasomes activation. Han et al*.* first demonstrated that emodin treatment (12.5–50 μM) inhibited the activation of NOD-like receptors (NLR) family pyrin domain containing 3 (NLRP3) inflammasome, leading to attenuation of cleaved IL-1β and caspase-1 secretion in LPS-induced bone marrow-derived macrophages (BMDMs) model [44]. It is well-characterized that IL-1β maturation and release are catalyzed by adenosine triphosphate (ATP)-mediated stimulation of P2X ligand-gated ion channel 7 (P2X7) receptor, which drives NLRP3 inflammasome assembly and activation [45]. Zhang et al. found that emodin (60 mg/kg) significantly inhibited the expression levels of P2X7 and NLRP3, thus delaying 5.0% sodium taurocholate-induced inflammasome activation and severe acute pancreatitis (SAP) progress in rats [46]. On the other hand, in an in vitro ATP‑injured model of human pancreatic ductal epithelial cell line (HPDE6‑C7), this protective activity of emodin against SAP was abrogated after pretreatment with P2X7 overexpression plasmid, which further verified that P2X7/NLRP3 signaling was the target of emodin on regulating inflammasome activation [47]. Moreover, a recent study revealed that emodin (25 mg/kg) protected against SAP-associated acute lung injury (ALI) in rats through the inhibition of NLRP3 inflammasome and uncovered a novel mechanism. By using nuclear factor erythrocyte-2 associated factor-2 (Nrf2) siRNA pool and ML385 to block Nrf2 transcriptional activity, Gao et al*.* suggested that Nrf2/HO-1 pathway was necessary for the protective effects of emodin, probably due to autophagy-mediated inhibition of NLRP3 inflammasome in response to Nrf2 signaling [48]. Xu et al*.* further determined that emodin (40 mg/kg) markedly mitigated SAP-upregulated cold-inducible RNA-binding protein expression and NLRP3 inflammasome formation to attenuate progressive pulmonary neutrophil infiltration in SAP-ALI rats, via deactivating NLRP3/IL-1β/CXCL1 signal [49]. Pyroptosis has recently been well-characterized as inflammasome-related programmed cell death and gasdermin D is defined as an executioner for pyroptosis downstream of inflammasome activation. As expected, a previous study found that emodin (20 mg/kg) alleviated myocardial ischemia reperfusion injury and gasdermin D-induced pyroptosis by blocking TLR4/MyD88/NF-κB/NLRP3 inflammasome pathway [50].

Several signaling cascades have been suggested to be involved in the fine-tuning of inflammatory responses and are potently regulated by emodin treatment. Functioning as critical immunological signaling molecules, Janus kinase (JAK) and signal transducer and activator of transcription (STAT) drive the expression of acute-phase proteins as well as a broad spectrum of inflammatory cytokines and are significantly implicated in the pathogenesis of a large variety of inflammatory and autoimmune diseases as well characterized. Emodin treatment (10 mg/kg) was reported to decreased serum levels of PCT, IL-6 and TNF-a, mitigated inflammatory response and alleviated jejunum damage, via de-activating JAK1/STAT3 signal, elevating the downstream target B-cell lymphoma 2 (BCL-2) and lessening BCL-2-associated X level in rats with sepsis [51]. Additionally, vascular endothelial growth factor (VEGF) is identified by its properties to induce severe vascular permeability and hypertonic pulmonary edema, hence its potential for a pathological role in ALI [52, 53]. Emodin (20 or 40 mg/kg in vivo and 10–40 μM in vitro) inhibited mammalian target of rapamycin (mTOR)/hypoxia-inducible factor-1alpha (HIF-1α)/VEGF signaling pathway and alleviated inflammation in rats with LPS-induced ALI and RAW264.7 cells [54].

Studies with respect to 11β-hydroxysteroid dehydrogenase type 1 (11β-HSD1) reported that its homozygous deletion not only suppressed inflammatory injury in adipose tissue, but also caused secondary effects on inflammatory mediator production by affecting immune cells like macrophages expressed 11β-HSD1 [55, 56]. As a potential 11β-HSD1 inhibitor, emodin at 10 μM modulated innate immune responses through down-regulating PTEN level and its downstream cytokines expression such as IL-6, IL-1β and TNF-α in LPS- treated 3T3-L1 adipocytes [57].

### Immunomodulatory effects

A well-functioning immune system depends on the homeostasis of both innate and adaptive immune responses involving a variety of immune cells such as macrophages, T cells, B cells, and neutrophils, and plays pivotal roles in maintaining normal physiological and immunological functions. As a highly heterogeneous population of innate immune cells, macrophages display proinflammatory or anti-inflammatory as well as immunoregulatory phenotypes in response to diverse microenvironmental signals, which are termed as classically activated macrophages (M1 macrophages) presenting pro-inflammatory phenotype and alternatively activated macrophages (M2 macrophages) producing anti-inflammatory mediators, respectively [58]. Several previous studies have reported that emodin modulated macrophage polarization and memory (Fig. 1). Iwanowycz et al*.* stimulated primary mouse macrophages with LPS/interferon gamma (IFN-γ) or IL-4, and found that emodin treatment at 50 μM bidirectionally tuned the stimulations and performed two-way regulation on macrophage polarization to restore homeostasis. Specifically, Emodin delayed M1 polarization via inhibiting NFκB/IRF5/STAT1 signaling pathways, whereas facilitated M2 alternative activation by suppressing IRF4/STAT6 signaling [59]. Concurrently, by applying microarray analysis, Iwanowycz et al*.* highlighted the involvement of epigenetic modifications in the effects of emodin on macrophage polarization. As suggested, emodin considerably and specifically changed the expression of several histone-modifying enzymes such as demethylase JMJD3 and KDM4A. Specifically, emodin inversely regulated a subset of genes involved in macrophage polarization, including M1-related TNFα, CXCL2 and CXCL10, as well as M2-related MRC1 and YM1 by preventing changes in H3K27 trimethylation (H3K27m3) and H3K27 acetylation (H3K27ac) at promoter regions of these specific key genes, without regulating genome-wide epigenetic profiles. Although these results are inspiring, further studies are urgently required to clarify the mechanism underlying the specificity of epigenetic modification.

Although it has been well-accepted that the transformation of M2 macrophages is beneficial for the relief of inflammatory injury, M2 macrophages are pathogenic in asthma by participating in T helper 2 (Th2)-associated immunity and are the key orchestrator of allergic asthma. The increased generation of Th2 cytokines (such as IL-4, IL-5) and antigen-specific IgE can be seen in the entire course of allergic asthma, followed by substantial eosinophil accumulation, pulmonary eosinophil infiltration, and mucus hypersecretion [60]. Emodin at 10 or 20 mg/kg significantly suppressed the number of total inflammatory cells and inflammatory mediator expression in the bronchoalveolar lavage fluid from ovalbumin-induced mouse model, supporting its therapeutic potential for treating allergic asthma [61]. Song et al*.* further found that emodin treatment at 2–50 μM alleviated these overactive immune responses via dose-dependently suppressing IL-4-evoked M2 macrophages polarization and STAT6 phosphorylation in BMDMs isolated from triple allergen (dust mice, ragweed, and aspergillus)-stimulated murine asthma model [16].

CD4^+^ cells, also known as “helper” cells, are a subpopulation of lymphocytes that lead the attack against infections by recognizing peptides presented on MHC class II molecules from antigen-presenting cells, and play a pivotal role in instigating and shaping adaptive immune responses. Generally, differentiated CD4^+^ cells subsets are characterized by specific cytokine profiles: T helper type 1 (Th1) cells by TNF-α, IFN-γ, and IL-2; Th2 by IL-4, IL-5, and IL-13; Th17 by IL-17, IL-6, and GM-CSF; regulatory T cells (Tregs) by IL-10 and IL-35 [62]. Immunomodulatory effects of emodin (100 µM) were in part attributed to its modulation of Th1/Th2 and Th17/Tregs balance via suppressing the release of Th1 and Th17 cytokines while inducing IL-4 and IL-10, which subsequently prevented concanavalin A-stimulated mouse splenocyte proliferation and autoimmunity [63]. Additionally, CD8^+^ (cytotoxic) T lymphocytes are also involved in adaptive immune response disorder-related diseases, including human chronic lymphocytic thyroiditis as well as experimental autoimmune thyroiditis [64]. It has been reported that emodin (75 mg/kg) effectively decreased the population of CD3^+^CD4^+^ and CD3^+^CD8^+^ T cells in peripheral blood mononuclear cell and spleen lymphocytes isolated from sodium iodide-induced experimental autoimmune thyroiditis mice model [65]. Emodin (10 mg/kg) was also confirmed as an emerging immunosuppressant that inhibited alloimmunity and alloantibody production by enhancing the production of both CD4^+^FoxP3^+^ and CD8^+^CD122^+^ Tregs, hindering dendritic cell maturation as well as blocking mTOR signaling-mediated inflammatory responses in C57BL/6 mice bearing allogeneic skin transplantation [66]. Furthermore, the highly conserved Notch signaling pathway, which regulates multiple steps of T- and B-cell development, is considered to have an emerging pattern of reciprocal regulation with inflammation [67]. A previous study reported that emodin at 60 mg/kg significantly mitigated airway inflammation in cough variant asthma mice by suppressing the Notch pathway, including Notch 1, 2, and 3, as well as delta-like ligand 4 expressions in lung tissue [68].

Neutrophils, the most plentiful type of white blood cells, are the frontier line of immune defense that travel through the bloodstream to the inflammatory lesions in need. Delayed neutrophils apoptosis and massive release of neutrophil extracellular traps (NETs) usually accentuate inflammatory response by inducing autoantigens production, which then trigger abnormal autoimmune responses and subsequently autoimmunity-related serious tissue injury, such as rheumatoid arthritis [69]. Recent evidence strongly supported that intraperitoneal injection of emodin at an extraordinary low dose (30 μg/kg/d for 6 days) efficiently prevented neutrophil infiltration and the release of pro-inflammatory cytokines like IL-6, IFN-γ, and TNF-α, and thus relieved rheumatoid arthritis symptoms as indicated by attenuated adjuvant-induced paw edema and ankle joint diameter, which was comparable to the positive control drug dexamethasone [70]. Further in vitro research also confirmed that emodin treatment (20 μM) significantly reduced BCL-2 and enhanced Bax and caspase-3 expression to reverse the resistance of LPS-primed neutrophil to phorbol 12-myristate 13-acetate-triggered apoptosis. The same dosage of emodin also inhibited NETs formation as well as NETosis in neutrophils isolated from murine adjuvant-induced arthritis model as illustrated by the downregulation of NET-associated myeloperoxidase and NE release. The authors suggested that these effects were probably depending on the disruption of autophagy since emodin significantly regulated autophagy-related genes expressions, such as BECN-1 and ATG5. In rats with SAP, emodin at 10 mg/kg was found to significantly suppress systemic inflammatory response syndrome by promoting neutrophil apoptosis via Ca^2+^/calpain-1/caspase-12/caspase-3 signaling pathway, while at 5 mg/kg mitigate pancreatic and intestinal mucosal injury by down-regulating caspase 1 [71, 72].

### Anti-fibrotic effects of emodin

**Fig. 2:**
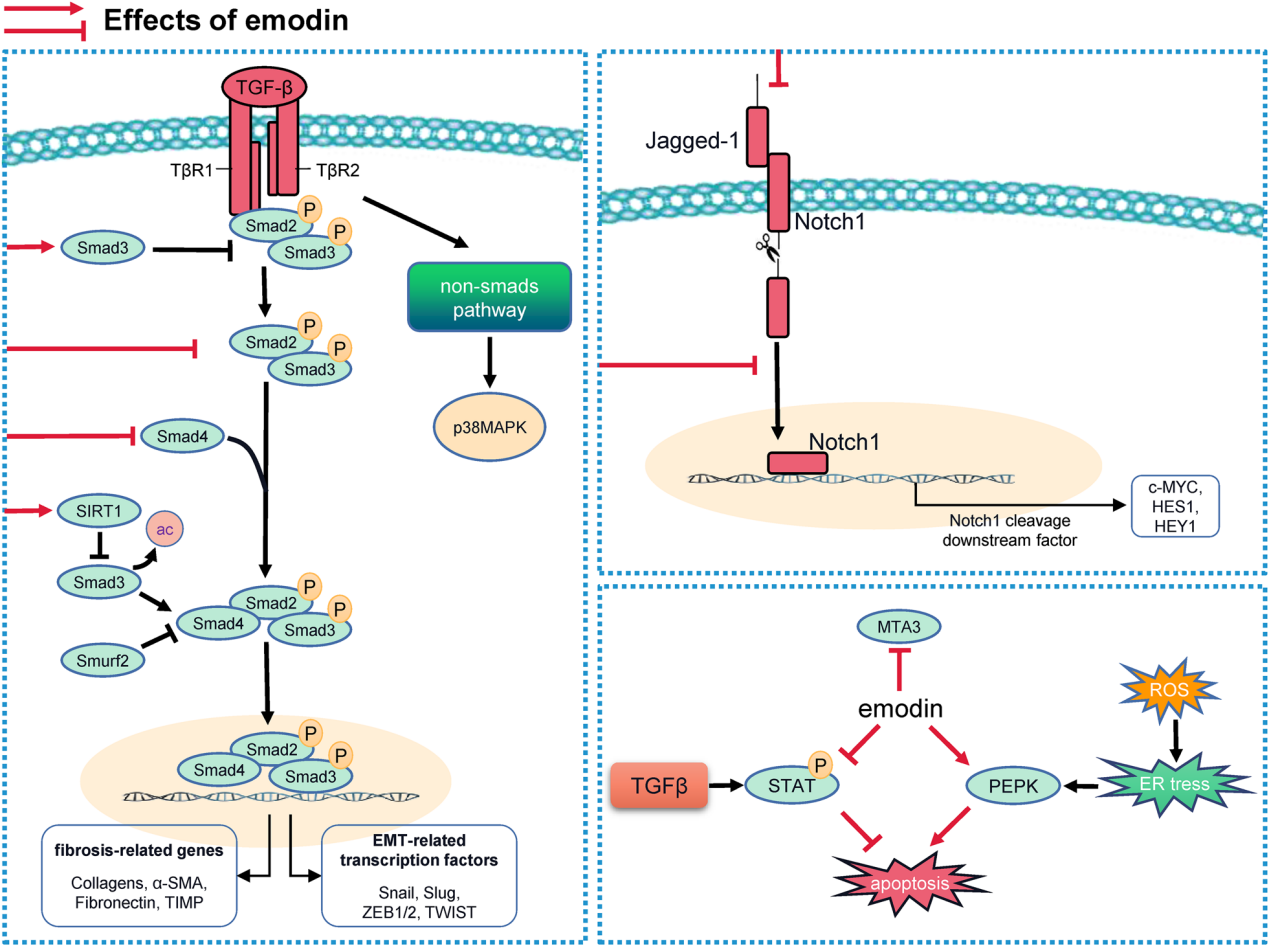
Networks of molecular signaling underlying anti-fibrosis property of emodin. Emodin inhibits EMT and organ fibrosis by blocking TGF-β/SMAD, p38MAPK signaling cascades as well as Notch pathway

Fibrosis, a hallmark of pathologic remodeling, virtually occurs in all organs and is characterized by the accumulation of extracellular matrix (ECM) proteins produced by activated fibroblast (myofibroblast) in response to persistent injury and unresolved inflammation [73]. Emerging evidence has identified that TGF-β signaling cascades, including both canonical downstream effectors small mothers against decapentaplegic (SMADs) as well as non-canonical (non-SMAD-based) signal, are master regulators during fibrogenesis, and are regulated by emodin (Fig. 2) [74]. In a rat model of liver fibrosis induced by carbon tetrachloride, mRNA expression of TGF-β1, and genes involved in its downstream signaling, including snail family transcriptional repressor 2 (SLUG), snail family transcriptional repressor (SNAIL), twist-related protein 1 (TWIST1), zinc finger E-box binding homeobox (ZEB)1 and ZEB2, and intrahepatic infiltration of Gr1^hi^ monocytes were profoundly decreased in emodin-treated group (10–40 mg/kg) [75, 76]. Interestingly, emodin (30 μM) was also reported to suppress ECM-related genes expression in hepatic stellate cells (HSC-T6) via blocking SMAD4 and p38MAPK signaling pathways, further suggesting a potential application of emodin for the treatment of liver fibrosis [77]. With high morbidity and dismal survival rate, pulmonary fibrosis remains one of the most devastating lung diseases, predominantly due to the absence of a valid biomarker for accurate diagnosis and limited therapeutic options. In bleomycin-induced pulmonary fibrosis rat model, emodin (20 mg/kg) alleviates fibroblast activation by repressing TGF-β1 expression and SMAD2/3 phosphorylation, and also preformed inhibitory effects on epithelial-mesenchymal transition (EMT) and ECM deposition in human alveolar epithelial A549 cells with similar mechanisms [78]. The silica inhalation-induced pulmonary silicosis mouse model was also established to investigate the effects of emodin treatment at 20 mg/kg on lung fibrosis, and the results showed that emodin increased the expression of Sirt1, which then deacetylated SMAD3 to inhibit TGF-β1/SMAD3 fibrogenic signal transduction, and thus attenuated collagen deposition [79]. With the help of siRNA-mediated Notch-1 depletion, Rundi Gao *et.al* suggested that emodin (2.5–20 μM) ameliorated TGF-β1-induced EMT and fibrosis in rat alveolar epithelial cells (RLE-6TN) via reducing Notch-1 nucleus translocation and suppressing Notch1/Jagged1 axis [80]. The same group further demonstrated that during the pathogenesis of idiopathic pulmonary fibrosis (IPF), infiltrated neutrophil-derived neutrophil elastase translocated into the nuclear area, cleaved nuclear Notch1, and facilitated the EMT process by inducing notch-dependent activation of C-MYC, HES1, and HEY1. Emodin at 10 μg/ml significantly inhibited the enzymatic activity of neutrophil elastase, which then inhibited nuclear notch-mediated EMT, and subsequently inhibited ECM production. These studies not only highlighted the critical roles of neutrophil infiltration and Notch1 signaling in IPF progression but also emphasized the plausible therapeutic effects of emodin for the treatment of IPF [81]. Furthermore, researchers also found that anti-fibrosis benefits of emodin (0.3 or 1 mg/kg) were related to down-regulation of SMAD ubiquitination regulatory factor 2 and up-regulation of Smad7 in surgery-induced renal fibrotic rats [82]. These studies have provided convincing evidence for the speculation that TGF-β signaling is the primary target of the anti-fibrotic effects of emodin.

Beyond the implications of classic TGF-β signaling pathways, it is also well-established that emodin prevents fibrosis by promoting fibroblast apoptosis and suppressing fibroblasts activation. STAT3 is suggested to be a latent transcription factor involved in survival and anti-apoptosis signaling and the aberrant activation of STAT3 induced by TGF-β is widely presented in tissue fibrosis [83]. Guan et al*.* suggested that emodin (15–60 μM) markedly suppressed expression of alpha-smooth muscle actin, collagen IV, and fibronectin in human embryo lung fibroblasts by abrogating the phosphorylation of STAT3, and thus alleviated pulmonary fibrosis in vivo [84]. Protein kinase R-like endoplasmic reticulum (ER) kinase (PERK) is critical in triggering ROS-mediated ER stress and mitochondrial apoptosis [85]. Emodin treatment at 50 or 100 mg/kg alleviated epidural fibrosis in rats after the laminectomy by stimulating fibroblast apoptosis via activating PERK signaling pathway and following ER stress [86]. Additionally, metastasis associated protein 3 (MTA3), a transcriptional co-regulator involved in the EMT process, suppresses fibroblast proliferation and migration and the silence of MTA3 expression was reported to result in the development of cardiac fibrosis [87, 88]. Recently, Xiao et al*.* elucidated that emodin at 20 μM significantly reversed the activation of cardiac myofibroblasts induced by AngII and at 40 mg/kg attenuated cardiac fibrosis in mouse model with pathological cardiac hypertrophy. Through gain- and loss-of-function analysis by using siMTA3 or Mta3-overexpressing plasmid, the authors identified that MTA3 was the key anti-fibrotic factor and was upregulated in the course of the beneficial action of emodin on cardiac fibrogenesis [89].

### Anti-cancer effects

#### Promotion of cancer cell apoptosis

**Fig. 3:**
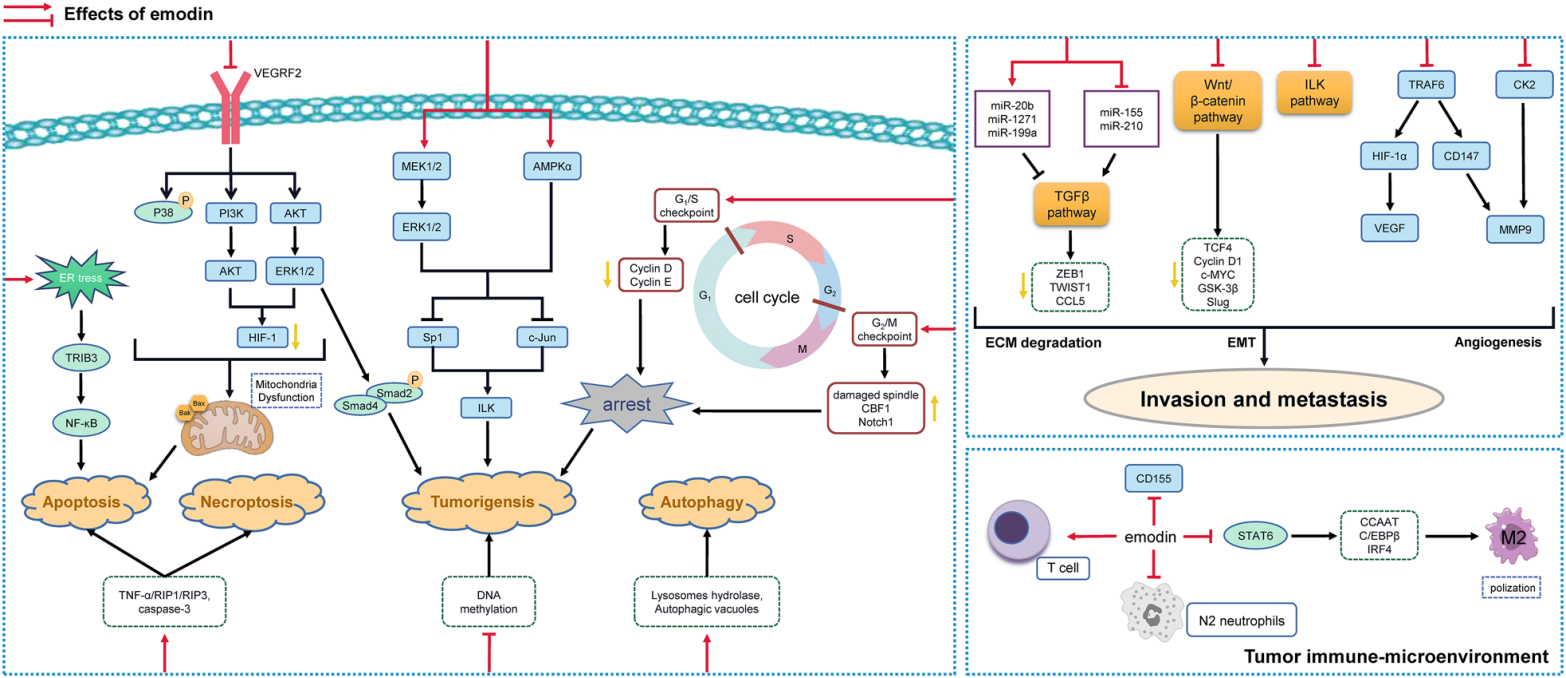
Molecular mechanisms underlying the anti-cancer effects of emodin. Emodin inhibits cancer growth through suppression of pro-survival signaling pathways, including p38MAPK, AKT, ERK, and ILK, and induction of cell cycle arrest and apoptosis. Emodin also blocks cancer invasion and metastasis by inhibiting EMT and pro-angiogenesis signaling, including TGF-β, Wnt/β-Catenin, ILK, VEGF, and MMP9. Emodin is also revealed to harness the immune system for curing cancer

A growing number of researches suggested that emodin could fulfill anti-cancer potential against various types of human malignant tumors through complex mechanisms (Fig. 3). Apoptosis is a physiologically programmed cell death process that removes damaged cells in an ordered and orchestrated form. Defects along apoptotic pathway take an essential part in carcinogenesis and numerous therapeutic strategies targeting apoptosis are feasible and effective in clinical practice [90]. Evidence has documented that emodin prevents cancer development by inducing apoptosis. Tribbles homolog 3 (TRIB3) is a pseudokinase protein and is known to aggravate ER stress-related cell apoptosis via NF-κB pathway [91]. Emodin treatment at 80 μM was found to activate TRIB3/NF-κB signaling and trigger ER stress-mediated apoptosis in human non-small cell lung cancer (NSCLC) cells (A549 and H1299) [92]. In addition, emodin (20–80 μM and 10–200 μM respectively) was reported to induce apoptosis of hepatocellular carcinoma (HCC) by suppressing PI3K/AKT and promoting the phosphorylation of p38 in HCC cell lines (SMMC-7721 and HepG2) [93, 94]. Emodin treatment at 50 or 100 μM was also found to induce mitochondria dysfunction and apoptosis in HepG2 cells by inhibiting pro-survival ERK phosphorylation and stimulating mitochondrial matrix-specific protein cyclophilin D expression [95]. Members of the BCL-2 gene family play a significant part in programmed cell death by regulating both pro-apoptotic and anti-apoptotic intracellular signals, and are fundamental to the balance between cell survival and apoptosis. It is well established that dysregulation of BCL-2 family genes results in apoptosis evasion in cancer by inducing mitochondrial outer membrane permeabilization and modulating downstream regulatory signals including MAPK/JNK, PI3K/AKT, NF-κB, and STAT pathways [96]. In the treatment of colon cancer, emodin (10–80 μM) was shown to decrease viabilities of human DLD-1 and COLO-201 cells through increasing the expression of pro-apoptotic BCL-2 family proteins Bax and Bak expression, and decreasing mitochondrial membrane potential in a time- and dose-dependent manner [97].

Autophagy is a highly regulated catabolic process associated with energy homeostasis and the basal turnover of cellular macromolecular precursors, and has bidirectional characters in cancer development, both pro-survival and pro-apoptotic, depending on certain circumstances [98]. A recent study revealed that emodin at 20 or 40 μM induced apoptosis in human colon cancer cell lines (HCT116 and LOVO) through a ROS accumulation-mediated autophagy activation [99]. Emodin (1–100 μM) also facilitated lysosomal-dependent autophagosome degradation via increasing the number of lysosomes and autophagic vacuoles as well as enhancing the activity of lysosomal hydrolases in cervical cancer cells (HeLa), and thus induced cell death [100]. Furthermore, necroptosis is another general mechanism of cell death independent from apoptosis. A previous study has found that emodin treatment at 0–40 μM could simultaneously induce apoptosis and necroptosis in human glioma cells (U251), by activating caspase-3 and TNF-α/RIP1/RIP3 signaling pathway, respectively [101]. However, specific mechanisms and potential targets were not elucidated.

#### Inhibitory effects of emodin on cancer cell proliferation

In addition to the induction of cancer cell death, most anti-cancer agents also perform significant anti-proliferative effects, which can be classified into two generic groups: anti-mitotic drugs and narrower anti-proliferative drugs [102]. Mechanistically, the cell cycle checkpoints such as G_0_/G_1_ and G_2_/M control cell cycle transition to limit the proliferation of cells bearing unstable chromosomes and to mediate cell dormancy [103]. Wang et al*.* demonstrated that emodin (15 μM) notably induced G_0_/G_1_ arrest in human gynecological cancer, including Hela cells, choriocarcinoma-derived (JAR) cells and ovarian cancer-derived (HO-8910) cells, through down-regulation of cell cycle regulators Cyclin D and Cyclin E [104]. Emodin treatment (1–100 μM) was reported to perturb mitotic progression by suppressing metaphase of mitotic process in Hela cells, manifesting as damaged spindle and accumulation of cells in G_2_/M phase [105]. Emodin (10–80 μg/ml) also induced G_2_/M arrest and restrained the proliferation of prostate cancer cells (PC3) via activating C-repeat binding factor 1 protein and subsequent Notch signal pathway [106].

Targeted therapy is a promising cancer therapeutics that specifically regulates the activity of oncogenes and tumor suppressor genes as well as the expression of proteins involved in the initiation and progression of malignancies. Tumor protein p53 is a tumor suppressor translated from TP53 gene, which is one of the most frequently mutated genes in human cancers. The misfolding and aggregation of p53 protein is detected as one of the frontiers during cancer development, and its accompanying risks disrupt the oncosuppressive function of wild-type p53 as well as some other related proteins like p63 and p73, which lead to malignancy. Emodin treatment at 15 or 20 μM was found to induce the disaggregation of these aggregates and restore the tumor-suppressive functions of p53 by increasing the autophagy levels in A549 cells [107]. Bioinformatics analysis also identified that emodin and its derivative E35 (10–40 μM) dose-dependently decreased levels of mutational p53 protein and the phosphorylation of PI3K/AKT to suppress proliferation and induced apoptosis of SU-DHL4 cell, a diffuse large B cell lymphoma cell line of Non-Hodgkin's Lymphoma [108]. Besides, miR-34a is a tumor suppressor microRNA transcriptionally activated by p53, and is identified as a crucial regulator of p53 function since it targets a considerable proportion of p53 network genes [109]. Emodin treatment at 10 or 100 nM suppressed HepG2 cells growth and inhibited SMAD2/SMAD4 expression by simultaneously inhibiting VEGFR2-AKT-ERK1/2 signaling pathway and promoting miR-34a level [110]. Similar results were also found in colon cancer cells and xenograft mice that emodin (20–80 mg/kg) suppressed colorectal cancer development by inhibiting the expressions of VEGFR2, PI3K, and p-AKT in xenograft mice model of HCT116 cells [111].

It is noteworthy that emodin also suppressed cancer cell proliferation via regulating targets other than P53 pathway. As one of the most popular anticarcinogen targets, HIF-1 mediates transcriptional activation of many genes coding proteins involved in key processes of cancer biology [112]. Emodin (20–200 μM) was found to inhibit HIF-1 biosynthesis, maintain energy homeostasis and exert anti-cancer effects via suppressing both PI3K/AKT and ERK signaling pathway in HepG2 cells [113]. Moreover, intracellular serine/threonine kinase integrin-linked kinase (ILK) is highly expressed in malignancies and recognized as a central regulator of signaling cascades that controls a series of biological processes in cancer progression, including but not limited to PI3K/AKT, Hippo, NF-κB, ERK and BCL-2 signals [114]. It has been suggested that the growth of NSCLC cells (A549, PC9, H1299, H1650, and H1975) was prevented by emodin (less than125 µM) since emodin reduced ILK expression by up-regulating MAPK extracellular signaling-regulated kinase (MEK)/ERK1/2 and AMP-activated protein kinase (AMPK)α signaling pathway, as well as decreasing the expression of specificity protein 1 (Sp1) and c-Jun protein [115].

Recent studies have further found that emodin yielded potential in epigenetic regulation. DNA methylation epigenetically participates in the process of gene expression and chromatin organization in normal cells, and has been confirmed to serve an essential role in tumorigenesis. Emodin at 0–80 µM suppressed the growth of pancreatic cancer cells (PANC-1) by inhibiting the expression of methyltransferase DNMT1, DNMT3a, and DNMT3b, which induced demethylation of the promoters of tumor-suppressor genes, including P16, Ras association domain family 1 isoform A (RASSF1A) and preproenkephalin (ppENK) and enhanced their transcription [116]. Moreover, a recent bioinformatic analysis further identified five differentially expressed genes (DEGs), including lysophosphatidic acid receptor 6 (LPAR6), complement C5 (C5), somatostatin receptor 5 (SSTR5), G protein-coupled receptor 68 (GPR68), and pyrimidinergic receptor P2Y4 (P2RY4). Emodin dose-dependently (25–100 μM) and markedly decreased the mRNA levels of these DEGs in HepG2 cells, suggesting those putative targets were likely to participate in emodin therapy for HCC, whereas the underlying molecular mechanisms remain to be verified by experimental evidence [117].

#### Effects of emodin on tumoral EMT and metastasis

As a primary hallmark of cancer progression, tumor metastasis is generally intertwined with drug resistance, poor prognosis and tumor relapse, and thus causes poor survival rates in patients with advanced cancer despite successful surgical resection or chemoradiotherapeutic treatment. Metastasis typically occurs following a complex succession of ECM degradation, EMT program, and angiogenesis. EMT, characterized by disappeared intercellular tight junction, reconstituted cellular backbone as well as changed cellular morphology, has been increasingly demonstrated to occupy a striking position in the process of carcinoma invasion and metastasis [118]. In response to angiogenic stimulus, tumor microenvironment is remodeled by ECM stiffness and degradation, then tumor cells exit primary lesions and enter the circulation by angiogenesis-provided principal route. Consequently, targeting the overlapping signals of these events helps to control tumoral EMT and metastasis coordinately, in order to potentially extend survival. As summarized above, the inhibitory effects of emodin on TGF-β-mediated EMT process in non-malignant diseases are promising, suggesting potential therapeutic activities of emodin against cancer cell EMT and cancer invasive growth. It was found that emodin at 20 or 40 µM suppressed EMT and invasive ability of pancreatic cancer cells (SW1990) by promoting miR-1271 expression and decreasing ZEB1 and TWIST1 expression post-transcriptionally [119]. Emodin (20–80 mg/kg) was also reported to possess both anti-angiogenesis and anti-EMT abilities in orthotopically transplanted mouse pancreatic cancer model of SW1990 cells by enhancing miR-20b expression and inhibiting angiogenesis-correlated TGF-β/SMAD pathway as well as levels of miR-155 and miR-210 [120]. Furthermore, Song et al*.* revealed a potential Forkhead box D3 (FOXD3) binding site within the promoter of miR-199a gene. Emodin at 20 µM directly promoted FOXD3-mediated miR-199a transcription, which in turn suppressed the translation of pro-fibrotic TGF-β2 to inhibit matrix metalloproteinases production and ECM degradation, and subsequently inhibited the invasive growth of A2780 cells [121]. Besides, fibroblasts isolated from the interface zones of triple negative breast cancer were found to induce EMT programs in co-cultured epithelial cells (BT20), which were reversed by emodin treatment (30 μM) through inhibiting TGF-β signaling pathway [122]. In addition to TGF-β-mediated EMT, peritumoral adipose tissue tends to interact with solid tumors and present a hospitable microenvironmental for invasiveness and dissemination by secreting cytokines and chemokines to induce the EMT process. Emodin (25–200 μM) was reported to prevent EMT by decreasing the secretion of CC-chemokine ligand 5 (CCL-5) from the co-culture system of adipocytes and triple negative breast cancer cell lines (MDA-MB-231 and MDA-MB-453) [123].

Several canonical proliferative signalings also contribute to the remodeling of tumor microenvironment and promote the EMT process and metastasis. The wingless-related integration site (Wnt)/β-catenin signaling plays vital roles in diverse biological processes such as the maintenance of epithelial cell phenotype, proper cell junctions, and tissue homeostasis, and it constitutively actives in diverse cancers to promote EMT and cell proliferation [124, 125]. Emodin treatment (5–20 µM in vitro or 40 mg/kg in vivo) blocked EMT and inhibited tumor growth through suppressing Wnt/β-catenin signaling downstream targets like TCF4, Cyclin D1, and C-MYC in human colon cancer cell line (RKO) as well as xenograft mice model, which was further abolished by agonist LiCl to confirm [126]. It is well known that the increase of glycogen synthase kinase 3β (GSK-3β) activity, followed by β-catenin phosphorylation, and the activation of ZEB1 are pivotal mechanisms promoting tumor invasion and metastasis. Hu et al*.* found that concomitant knockdown of ZEB1 expression and emodin treatment (20 µM) synergistically suppressed cell invasion and EMT of epithelial ovarian cancer cells (A2780 and SK-OV-3) to a greater degree via suppressing Wnt/β-catenin signaling [127]. Besides, the regulative effects of emodin on ILK pathway also contribute to the anti-EMT effects of emodin. Recent studies in A2780 and SK-OV-3 cells claimed that emodin restrained the migration and invasion in ovarian cancer by reducing ILK expression. Emodin further abrogated the phosphorylation of ILK downstream target kinases, GSK3β and Slug [128, 129].

Notably, TNF receptor-associated factor 6 (TRAF6), a member of TRAF protein family that transduced signals from TNF receptors, inhibits HIF-1 polyubiquitination-mediated degradation and thus promotes tumor angiogenesis [130]. Besides, as another downstream regulator of TRAF6, extracellular matrix metalloproteinase inducer (CD147) mediated matrix metalloproteinases (MMPs) overexpression to favor extracellular matrix digestion and metastasis [131]. Emodin (less than 40 µM) dose-dependently suppressed both TRAF6/HIF-1α/VEGF and TRAF6/CD147/MMP9 signaling pathways to simultaneously inhibit pro-angiogenesis and invasion capacity of human anaplastic thyroid cancer cell lines (8505c and SW1736) [132]. In support of this study, the VEGF and VEGFR-2 protein levels were profoundly decreased after emodin treatment at 15 μM in Hela, JAR, and HO-8910 cells [104]. It was also demonstrated that emodin (5–200 µM) repressed MMP-2 and MMP-9 expression to restrain the migration and invasion of HCC MHCC-97H cells, by activating p38MAPK signal and inhibiting ERK/MAPK and PI3K/AKT signals [133]. Furthermore, as a CK2 inhibitor, emodin (20 µM) was revealed to suppress protein kinase C-induced tumor cell invasion through restraining CK2 activation and subsequently decreasing MMP-9 expression in breast cancer cells (MCF-7) [134].

#### Effects of emodin on tumor immune-microenvironment

The antineoplastic properties of emodin are also linked to cancer immunotherapy. The interaction between tumor cells and macrophages can create a feedforward loop and trigger M2-macrophage polarization, which in turn supports tumor growth and migration via establishing an immunosuppressive tumoral microenvironment [135]. As speculated, the modulatory effects of emodin on the immunosuppressive tumor microenvironment are largely dependent on its immunoregulatory activities and share similar mechanisms. Iwanowycz et al*.* pointed out that emodin (40 mg/kg) reversed M2-macrophage polarization by suppressing STAT6, cytidine-cytidine-adenosine-adenosine-thymidine (CCAAT)/enhancer-binding protein β (C/EBPβ) as well as IRF4 signaling, enhanced T cell activation, and thus blocked breast cancer growth and metastasis in mice bearing EO771 or 4T1 tumors [136]. Additionally, recent evidence further suggested that short-term administration of emodin (40 mg/kg) for 10 days before mastectomy surgery effectively suppressed EMT and cancer stem cell formation, and thus harbored the potential to halt metastatic recurrence of breast cancer, via blocking TGF-β1-mediated crosstalk between tumor-associated macrophages and cancer cells [137]. The adhesion molecule CD155 is highly expressed in various types of tumors and is associated with cell motility and immune evasion from natural killer cell and T cell-mediated cytotoxicity [138]. Emodin (20 or 50 μM) notably suppressed the proliferation and migration of mouse EO771 and 4T1 cells as well as B16-F10 melanoma cells by down-regulating their expression of CD155 [139]. Moreover, N2-like neutrophils (HL-60N2, CD66b^+^) are proposed as the pro-tumoral paradigm and serves an essential role in hypercoagulation and cancer progression by contributing to immunosuppression and NET [140]. Emodin (10 mg/kg) selectively suppressed high numbers of N2 neutrophils and NETs in lung and prevented urethane-induced hypercoagulation and lung carcinoma lesions in ICR mice model [141].

#### Anti-tumor effects of emodin in combination with other therapies

The anti-tumor benefits of emodin in combination with other antineoplastic drugs have been extensively explored recently. Several pharmacodynamics studies have reported that emodin could sensitize tumor cells to chemotherapies and confer notable synergistic actions. Sorafenib, by targeting multiple tyrosine kinases required for tumor growth and metastasis, is currently the first-line therapy available for advanced HCC [142]. It has been demonstrated that a combination of emodin (20 μM) and sorafenib (2 μM) has an additive effect to inhibit tumor growth through decreasing AKT and STAT3 oncogenic growth signaling in mice model xenografted with HCC cells (HepG2 or SK-HEP-1) [143]. Emodin (10 μM) also synergistically sensitized A549 cells towards paclitaxel (4 μM) through inhibiting cells proliferation and stimulating apoptosis. The mechanisms were found to be associated with the promotion of Bax and caspase 3 expression, down-regulation of BCL-2, as well as suppression of AKT/ERK pathway [144]. Notch-regulated ankyrin repeat protein (NRARP) is a key regulator in both Notch and Wnt signaling pathways that are essential for breast cancer proliferation [145]. Emodin (20 µM) potentiated 5-fluorouracil (5-FU)-based (40 µM) cellular senescence and apoptosis in MCF-7 cells by silencing NRARP, which was reverted by NRARP overexpression [146]. Emodin (less than 80 µM) in combination with demethylation agent 5-Aza-2'-deoxycytidine (5-Aza-CdR) (1 μM) synergistically inhibited the growth of PANC-1 cells in a dose- and time-dependent manner and enhanced the demethylation of p16, RASSF1A, and ppENK through suppressing the expressions of DNMT1 and DNMT3a [147].

The development of resistance to conventional chemotherapies and target therapies represents the major challenge for cancer treatment, and emodin has been reported to yield the potential to enhance the therapeutic effectiveness of chemotherapies as an effective adjuvant agent circumventing drug resistance. Epidermal growth-factor receptor (EGFR) overexpression is closely related to the progression of pancreatic cancer, while drug resistance of EGFR inhibitors (such as afatinib) limited its clinical application [148]. It was found that emodin treatment at 30–90 μM markedly reversed afatinib (20 nM) resistance and promoted apoptosis via suppressing STAT3 and EGFR expression in pancreatic cancer cells (PANC-1 and BxPC-3) [149]. The anti-neoplastic activities of anthracyclines such as daunorubicin (DAUN) and doxorubicin (DOX) are limited due to the resistance and cumulative cardiotoxicity of their reduction products. Emodin was identified as a potent inhibitor for anthracycline reductases, and the DANU resistance was confirmed to be reversed by emodin at 30 μM in A549 and HepG2 cells [150]. Multi-drug resistance-associated protein (MRP), P-glycoprotein (P-gp), ATP binding cassette sub-family G member 2 (ABCG2) and other efflux transporters have been found to pump out various chemotherapeutic agents, and are considered to be predominant causes leading to drug resistance and therapy failure. Emodin at 15 or 20 μM respectively potentiated cisplatin-induced (1 or 1.5 μg/ml) cytotoxicity against human bladder cancer cell lines (J82 or T24) through elevating cellular ROS level and decreasing MRP1 expression [151]. Emodin (10 or 50 μM respectively) also potentiated the DOX-induced (3 or 7.5 μM) growth inhibition of A549 and colon carcinoma cells (HCT-15) and prevented the development of DOX resistance through increasing low-density lipoprotein receptor-related protein 1 expression and suppressing the activity of efflux transporters like P-gp, ABCG2 and MRP1-4 [152]. The effects of emodin on the regulation of other MDR-related transporters and underlying mechanisms remain to be clarified.

### Anti-viral activity of emodin

The general aim of antivirals is to prevent virus load from increasing to a point where virus infection is established, and they often act by arresting the viral replication cycle at nearly the whole steps in the virus life cycle ranging from entry to release. Recent years have witnessed growing evidence that emodin possesses favorable anti-virus effects and is frequently used for the treatment and prevention of epidemic diseases caused by viruses. The lytic replication of Epstein-Barr virus (EBV) has been found to be involved in the pathogenesis of NPC as a causative agent. Emodin treatment (1–50 μM) significantly inhibited EBV lytic proteins expression and virion production in EBV-positive NPC cell lines NA and HA, through interfering with the protein expression of Sp1 binding to Zta promoter (Zp) and Rta promoter (Rp) [18]. Moreover, it was also demonstrated that emodin (40 µM) could significantly inactivate Zika virus and attenuate its entry as well as infectivity in Vero E6 cells by directly impairing viral particles [153]. Furthermore, emodin (25 μg/ml) inhibited influenza A virus replication and mitigated influenza viral pneumonia through activating Nrf2 signal and inhibiting ST169- and H1N1-induced oxidative stress, inflammation, as well as elevation anti-viral mechanisms, including TLR4, p38/JNK MAPK, and NF-κB pathways, in Madin-Darby canine kidney cells and A549 cells [154].

Enterovirus 71 (EV71) and coxsackievirus B3m (CVB3) are primary causative agents of hand-foot-mouth disease (HFMD) causing neurological and systemic complications, and therefore, pose a substantial burden to parents and caretakers across the Asia–Pacific region and beyond. Due to the absence of effective vaccines, the best prevention for HFMD is avoiding contact with infected individuals, and therefore, new therapeutic drugs for EV71 or CVB3 have attracted huge attention and are urgently needed. Previous studies demonstrated that emodin (29.6 μM) directly inhibited EV71 maturation to protect host cells from cytopathic effects, through suppressing the efficiency of genome replication and protein expression of the virus via diminishing cell cycle arrest of host cells at S phase [155]. Ding et al*.* further reported that emodin (20–80 mg/kg) possessed a protective property on CVB3-mediated HFMD mice model through decreasing TLR3 expression as well as downstream targets, including TRIF, TRAF3, TRAF6, IRF3, NEMO, NF-κB, TBK1, TAB3, IKK-ε, MAPK and AP1 [17]. Emodin (20 μM) also restrained the translation and synthesis of CVB3 viral protein 1 and conferred the host with resistance to virus infection via multiple pathways in HeLa cells and HL-1 cardiomyocytes infected with CVB3, including blocking AKT/mTOR signaling, activating eukaryotic initiation factor 4R-binding protein 1 and eukaryotic elongation factor 2 kinase [156].

### Anti-bacterial effects of emodin

Bacterial biofilm refers to the densely packed communities of microbes, which assists bacteria adapt to harsh conditions and keeps them less susceptible to antimicrobial treatments [157]. Emodin (1/8 MIC-MIC) decreased the biofilm growth of *Streptococcus suis* strain ATCC700794 and *Staphylococcus aureus* strain CMCC26003. Among them, its antibacterial mechanism on *S. aureus* strain CMCC26003 was related to intervening the release of extracellular DNA and down-regulating the expression of the biofilm-related genes such as cidA, icaA, dltB, agrA, sortaseA, and sarA [158, 159]. Methicillin-resistant *S. aureus* (MRSA) is a major type of gram-positive bacterium leading to nosocomial infection. There is evidence suggesting that several MRSA strains emerge and exhibit growing antimicrobial resistance to vancomycin and linezolid. Emodin (2–32 μM) exerted in vitro anti-bacteria activities against standard *S. aureus* strain 252 (MRSA252) and 36 MRSA clinical strains via disrupting the integrity of cytoderm and cytomembrane of MRSA. It is noteworthy that emodin (0.5–32 μg/ml) remarkably inhibited MRSA infection without showing significant cytotoxicity against mouse RAW264.7 cells [160]. The proteomic analysis further elucidated that emodin (3.9 mg/ml) was performed as a potential amino nucleoside antibiotic to inhibit MRSA133630 growth. The associated antibacterial mechanisms were suggested as down-regulating pyruvate kinase as well as chromosome glycolytic, and suppressing ribosome- and Aminoacyl tRNA synthetase-dependent errant protein translation [161].

In addition to directly targeting bacteria, the protective effects of emodin also attribute to the modulation of host cell responses. *Haemophilus parasuis* is a symbiotic bacterium of swine upper respiratory tract and performs as the etiological risk factor of Glasser's disease. Emodin (32 or 64 μg/ml) was identified as a candidate for treating Glasser's disease due to its dose-dependently inhibitory effects on serious host cell injuries, including increased membrane permeability, plasmolysis, and cytoplasmic vacuolation [11]. The isobaric tag for relative and absolute quantification (iTRAQ)-based quantitative proteomic analysis was applied by Li et al*.* to investigate the antibacterial mechanism of emodin on *H. parasuis* HS80 strain, and revealed that emodin at 16 μM suppressed the adhesion and invasion of HS80 in porcine kidney cells (PK-15) [162]. Moreover, as a food supplement, emodin (0.1–0.4 g/kg) potentiated the specific and nonspecific immune defense of walking catfish against *Aeromonas hydrophila* infection by enhancing the activity of SOD and lysozyme and elevating phagocytic activity in head-kidney leucocytes [163].

### Effects of emodin on diabetes and complications

Emerging evidence suggested that nutrient absorption, especially glucose uptake, was potentially regulated by emodin. In line with previous reports, a recent study confirmed that emodin (3.125–12.5 µM) dose-dependently enhanced the insulin-provoked glucose uptake in IR HepG2 cells based on 2-NBDG glucose uptake assay [164]. The intestinal α-glucosidase (AG) is responsible for the digestion of complex food-sourced polysaccharides and its activity constrains calories intake. The upregulation of AG may lead to an abnormal rise in blood glucose, especially in diabetes, by promoting rapid disaccharides hydrolyzation and glucose absorption. Emodin exhibited intestinal AG inhibitory effects with an estimated IC_50_ of 30 μg/ml and controlled postprandial spikes of blood glucose by directly interacting with Ser74 binding site, as indicated by molecular docking [165]. Moreover, studies showed that a T-cell surface marker CD26, also named dipeptidyl peptidase 4 (DPP-4), is considered to be a novel adipokine that potentially impairs glucose tolerance and insulin sensitivity in both an autocrine and paracrine fashion [166]. DPP-4 inhibitors, such as BI 1356 and linagliptin, are effective in the treatment of type 2 diabetes and have entered into the clinical research, as they effectively maintain blood glucose levels through degradation of incretin peptides, glucagon-like peptide 1, and glucose-dependent insulinotropic polypeptide. Emodin was further identified as a small molecule inhibitor of DPP4 with an IC_50_ of 5.76 μM by forming H-bonds with Glu205 and Glu206 at DPP4 active site as predicted by the docking model. In vivo study further confirmed that 3–30 mg/kg orally administrated of emodin significantly decreased peripheral DPP4 enzymatic activity, suggesting emodin as a potential therapeutic candidate for diabetes [167].

The regulation of metabolism homeostasis in peripheral tissues, such as liver, adipose, and skeletal muscle, is critical for the maintenance of insulin sensitivity and for the treatment of metabolic syndrome. There are growing experimental evidence and clinical data suggesting that emodin treatment alleviates IR and MS-related diseases, by modulating multi-tissue glucose and lipid metabolism. The nutrient sensor AMPK is a crucial receptor and regulator of energy balance maintaining glucose and lipid homeostasis. Emodin (0.25 or 0.5 μg/ml) was reported to attenuate nonalcoholic fatty liver disease (NAFLD) in zebrafish by activating AMPK pathway, via improving PI3K/AKT2/AMPKα-mediated IR and enhancing AMPKα/PPARα/CPT1 and AMPKα/PPARα/ACOX1-mediated fatty acid oxidation [168]. Similarly, Emodin (6.25–50 μM) remarkably activated glycolysis and suppress lipolysis in C2C12 myotubes and 3T3-L1 adipocytes by up-regulating AMPK signal and down-regulating perilipin level [169]. Sterol regulatory element-binding proteins (SREBPs), the key factors of cellular lipid metabolism and homeostasis, participates in the transcriptional regulation of genes involved in lipid biosynthesis and uptake, including ACACA, FASN, FABP, HMGCR, HMGCS, and several studies reported that AMPK negatively regulates SREBP to limit lipogenesis under nutrient deficiencies by directly phosphorylating SREBP. Studies have found that emodin (40, 80, and 160 mg/kg) effectively inhibited SREBP1c activation to improve lipid accumulation and ameliorate hepatic steatosis in NAFLD rats, via suppressing liver endoplasmic reticulum stress and activating calcium/calmodulin-dependent kinase kinase-AMPK-mTOR-p70S6K-SREBP1 signaling pathway [170, 171]. Emodin at 40 or 80 mg/kg also improved high fat diet (HFD)-induced obesity and metabolic disturbances by reducing the expression of both SREBP1 and SREBP2 in the liver and adipose tissue of HFD mice, which further inhibited lipid biogenesis and uptake [172, 173]. Furthermore, emodin (20–80 mg/kg or 20 μM) was found to improve glucose metabolism and decrease lipid accumulation in HFD metabolic syndrome model by down-regulating miR-20b and activating its target gene SMAD7 in IR skeletal muscle both in vivo and in vitro [174]. Another analogous study further reported that emodin at 25 or 100 mg/kg ameliorated lipid accumulation in soleus muscle of HFD rats by inhibiting fatty acid transport proteins 1-mediated fatty acid transmembrane transportation and lipid accumulation [175]. Interestingly, a most recent study found that emodin treatment (40 and 80 mg/kg) markedly induced beiging of white adipose tissue and brown adipose tissue activation by up-regulating the expression of CD36 and FATP4, thus accelerated the transport and consumption of fatty acids and improved obesity [176].

However, Abu Eid et al*.* illustrated that the anti-hyperglycemic activity of emodin treatment (103 and 229 mg/kg) in obese mice was driven by its side effect on diminishing appetite but not regulative effects on metabolism. Specifically, along with the reductions of food consumption, body weight gain and adiposity, emodin surprisingly decreased insulin release, desensitized insulin responses, and deteriorated glucose tolerance in obese mice, which were contrasted with its widely recognized anti-diabetic effects [177]. This observation not only indicated the plausible risk of prolonged use of emodin in diabetic patients but also highlighted that the putative therapeutic effects of emodin on metabolic syndrome, including fatty liver diseases or diabetes, need to be further investigated and confirmed.

Emodin also plays a significant role in preventing and alleviating diabetes complications such as diabetic cataracts, diabetic nephropathy (DN), and diabetic peripheral neuropathy. As a critical enzyme of the polyol pathway, aldose reductase (AR) catalyzes the reduction process of glucose to sorbitol and can be activated to induce the development of diabetic complications under hyperglycemic states [178]. It has been implicated that emodin at 5 μM (in vitro) or 35 mg/kg (in vivo) possessed selectively inhibitory activity against AR in human lens epithelial cells (FHL124) and reduced the incidence of cataract morphological pathological changes like vacuole formation and lens opacification in AR transgenic mice (strain PAR37) model. This potent biological activity was dependent on the 3-hydroxy group of emodin, which formed a tight hydrogen bond with Ser302 in the specificity pocket of AR [19]. An increasing body of evidence demonstrates that sustained or aberrant ER stress contributed to the pathogenesis of DN by mediating multiple signals including PERK/eukaryotic initiation factor 2α pathway. Tian et al*.* claimed that emodin treatment (40 or 80 mg/kg) dose-dependently decreased urinary albumin and improved the renal function and pathological changes such as mesangial matrix expansion and hyperplasia of collagen fibers in KK-Ay mice with DN. Emodin (20 or 40 μM) was also found to alleviate ER stress and podocytes apoptosis in mouse podocyte cell line (BNCC337685) stimulated with high glucose via suppressing PERK/eIF2α signaling pathway [179]. Besides, emodin at 100 mg/kg protected against DN and improved tubulointerstitial injury through inhibiting the activity of ICAM-1, pro-apoptotic factor Bax, and caspase-3, as well as promoting PI3K/AKT/GSK-3β pathways in rat model [180]. Emodin at 10–40 µM even inhibited the proliferation of aggravated renal mesangial cell (RMCs), which was another pathological character in the early onset of DN, and stimulated cell cycle arrest at G_1_ phase by rising levels of Bax, caspase-3, cleaved caspase-3, caspase-6 and caspase-8 in rat RMCs line (HBZY-1) [181]. Methylglyoxal (MGO) is a byproduct of glycolysis, which leads to the formation of advanced glycation end products (AGEs), accumulating in the vessel wall and contributing to organs and tissues dysfunction and subsequently to clinical end points of debates with microvascular and macrovascular complications [182]. Both MGO scavenging and MGO-modified bovine serum albumin formation assays were performed and revealed that emodin (5–250 µg/ml) was a promising MGO scavenger and suppressed subsequent AGE accumulation in diabetic renal tissue, which alleviated proteinuria and podocyte loss in DN [183]. Unlike DN, the study of emodin in the treatment of diabetic-associated neuropathy is limited. A recent study reported that emodin (10 μM) protected neuron-like cell line (PC-12) against high glucose-triggered apoptosis and autophagy via upregulating miR-9 expression, activating PI3K/AKT and restraining NF-κB pathways [184].

## Toxicity of emodin

The potential toxicity of emodin, such as hepatotoxicity, nephrotoxicity, and reproductive toxicity, has been reported in recent years. There is growing evidence suspecting that emodin may act as the main causative agent in organ damage associated with rhubarb, *P. multiflorum*, *C.* semen, and other herbal medicines, while the exact mechanisms underlying emodin-induced cytotoxicity remain undefined. Yang et al*.* tested the cytotoxicity of emodin on human hepatic progenitor cells (HepaRG) and compared it with other eight major components in the aqueous extract of *C.* semen, and then confirmed that emodin was the most potential hepatotoxic phytochemicals in *C.* semen [13]. Recent research based on quantitative proteomics indicated that emodin-induced hepatocyte apoptosis by interrupting oxidative phosphorylation signaling pathway. Consistently, the function of the mitochondrial respiratory chain complex in L02 cells was inhibited after emodin treatment at 6.125–50 μM, which consequently led to an increase in caspase-3 and ROS, decrease in mitochondrial membrane potential, as well as disorders in ATP synthesis [20]. Zhang et al*.* further demonstrated that emodin (150 mg/kg) induced oxidative stress in rat livers via directly targeting Acadvl and complex IV as well as interfering with fatty acid β-oxidation, tricarboxylic acid cycle, oxidative phosphorylation, and ATP production [185]. Another study established 1H NMR-based metabonomic techniques to assess emodin toxicity in HepG2 cells. The levels of several physiological metabolites in both cell extracts and cell culture media were significantly altered by emodin treatment, and eight associated biological processes were enriched, such as Krebs cycle, amino acid metabolism, and purine metabolism, which were speculated as potential toxicity mechanisms and were similar with other findings [186]. Additionally, it was also reported that emodin at 40 or 80 μM showed nephrotoxicity by inducing apoptosis of human proximal tubular epithelial cells (HK-2) as well as interfering PPARγ-related mitochondrial pathway [21]. Emodin was also reported to possess reproductive toxicity. Both intracellular Ca^2+^ concentrations and tyrosine phosphorylation exert central roles in the regulation of sperm function. Emodin treatment (50–400 μM) dose-dependently interfered with these two mechanisms, and thus inhibited sperm total motility as well as their ability to penetrate viscous medium [187].

## Modification of emodin for better therapeutic potential

### Approaches to improve the poor water solubility and pharmacokinetic properties of emodin

Emodin is known to be instantly soluble in DMSO, ethanol, or alkali solution, but nearly does not soluble in water. Over the years, in vitro and in vivo studies on emodin have been limited due to its poor aqueous solubility, which brought challenges for further evaluation and interpretation of its pharmacological activities or toxicities, and created obstacles to novel drug development based on emodin. Generally, emodin exerts pharmacological activities at the concentration of a few tens of μM level, while poorly soluble emodin is easy to precipitate in the medium and leads to challenges in accurately determining the actual effective dose of drugs. In addition to poor water solubility, significant first-pass elimination of emodin in liver and intestine contributes to its limited oral bioavailability [188]. However, the pharmacokinetic properties and metabolism of emodin were not extensively characterized until now. Qin et al*.* identified three hydroxylation metabolites by LC–MS/MS analysis in rat and human liver microsomes incubated with 50 μM emodin, including 2-hydroxyemodin, 5-hydroxyemodin, and ω-hydroxyemodin. They also identified that human P450 enzymes 1A2 and 2C19 were the primary enzymes mediating the bioactivation of emodin [189]. Recently, 14 phase I metabolites derived from hydroxylation, hydrolysis, oxidation, or reduction of emodin were identified and could be detected in real-time by means of UPLC-Q-TOF/MS [190]. It is also noteworthy that the metabolism and biotransformation pathways of emodin after oral administration were strongly related to intestinal flora. Emodin and its ten metabolites were identified in human intestinal flora samples as a result of different metabolic pathways, including but not limited to reduction, hydroxylation, acetylation, demethylation, and methylation [191].

Numerous attempts have been made to improve the solubility and bioavailability of emodin to enhance its therapeutic potency. Pharmaceutical cocrystals is a form of molecular complexes consisting of candidate molecules and one or more cocrystal formers interacting through hydrogen bonds. Recently, Park et al*.* proposed and identified an emodin-nicotinamide (1:2) cocrystal, where emodin dimers connected alternatively with nicotinamide tetramers to form one-dimensional chains and two-dimensional layers sequentially. Compared with emodin, this novel cocrystal showed improved aqueous solubility, dissolution rate, and chemical stability [192]. It is worth noting that topical application of emodin is an effective complementary therapy to the oral route, since the latter showed low bioavailability due to its poor solubility and extensive first-pass glucuronidation [193]. Solubilizer or surfactant is another simple and direct option to improve solubility, and has long been used as critical excipients for topical drug delivery. Emodin-loaded thermo-reversible poloxamer gel, an attractive topical formulation composed of P407/P188/PEG400 (10/30/5), was modified to achieve improved drug compliance and bioavailability, leading to nearly 100-fold enhancement in emodin solubility compared to water [194]. Another transdermal drug delivery system based on Layer-by-Layer films assembled by poly(ethyleneimine) and poly(vinyl sufonate) was further developed, by immobilizing emodin directly in films or incorporating emodin in liposomes before intercalated, to mediated controlled and stabled release of emodin in vitro [195]. Similarly, ion-complementary self-assembling peptides were found to have potential in delivering hydrophobic drugs in a manner of sustained release. Under mechanical stirring, a preliminary suspension in-situ hydrogel delivery system of emodin was constructed with the self-assembling peptides RADA16-I and RVDV16-I, showing promising emodin loading and releasing properties, and performed better anti-cancer activities against A549 and HepG2 than free-emodin [196]. Besides, an emodin self-microemulsifying platform was designed and achieved improved solubility and better oral absorption in vivo as determined by pharmacokinetic assay. Based on this drug delivery system, emodin treatment attained better inhibitory effects on renal fibrosis both in rat model and in AGEs-treated glomerular mesangial cells and renal tubular epithelial cells, by suppressing the protein expression of fibronectin, TGF-β1 and ICAM-1 [197].

Efforts have also been made to improve the pharmacokinetic properties of emodin. With a β-OH on the anthraquinone ring, emodin acquired high glucuronidation activities and primarily transformed into emodin glucuronide with exceedingly low absorption and bioavailability, catalyzed by UDP-glucuronosyltransferases (UGTs) 1A1 and UGT1A9 [198, 199]. Zhang et al*.* developed an emodin nanoemulsion containing cremophor EL, the emulsifier worked as an effective glucuronidation inhibitor to block UGT1A1-mediated emodin glucuronidation. Accompanied by a substantial decrease (57.4%, *p* < 0.001) in total emodin glucuronidation, the apical and basolateral excretion of glucuronidated metabolite were markedly reduced (≥ 56.5%, *p* < 0.001) in UGT1A1-overexpressed MDCKII cells, and thus enhanced transcellular permeation and absorption of emodin [200].

### Other approaches to improve the therapeutic effects of emodin

From the standpoint of drug development, structural modification is a bright new avenue to improve physicochemical, biochemical, and pharmacokinetic properties, and to increase drug potency and selectivity. Emodin was recognized as a promising precursor compound in cancer therapy for the past few years. An innovative series of emodin derivatives were designed and modified, and their anti-proliferative and pro-apoptotic effects were assessed in HepG2, MCF-7, Hep3B, Huh7, A549, and various leukemia or lymphoma cell lines, which were well-summarized in a previous dedicated review [201]. One of these derivatives (compounds 7a) exhibited a potent in vitro anti-proliferative activity profile, displaying an 8.8-fold increase of IC_50_ value than emodin, by selectively inducing apoptosis and cell arrest at G_0_/G_1_ phase [202]. Recently, emodin succinyl ester was synthesized to inhibit the migration and invasion of HCC cells by restraining the interaction of androgen receptor and enhancer of zeste homolog 2. In vivo tumorigenicity tests using xenograft and diethylnitrosamine-induced HCC mouse model further validated its significant efficacy in preventing HCC aggression [203]. Viewed from the metabolic perspective, ATP citrate lyase, a key enzyme responsible for generating cytosolic acetyl-CoA, was overexpressed in cancer cells to regulate stemness and metastasis. Structure–activity relationship and docking analysis characterized a novel class of ATP citrate lyase inhibitors bearing the chemical scaffold of emodin, and indicated that two free hydroxyls adjacent to 9-carbonyl were critical for on-target activity, and halogens at 2- and 4-position further enhanced inhibitory activity. It was further demonstrated that 3,5-bisbromoemodin notably reduced cancer stemness in the 3D spheroid assay, and showed a comparable anti-proliferative activity against A549 cell to shRNA-mediated ATP citrate lyase knockdown, therefore making it an encouraging prodrug for tumor therapy [204]. Most recently, Chen et al*.* synthesized another class of emodin quaternary ammonium salt derivatives, and identified E35 (BrNO5·H2O) as a potential therapeutic candidate for hematologic cancers with higher aqueous solubility and specificity than emodin. E35 efficiently suppressed growth and enhanced apoptosis of various acute leukemia stem and progenitor cells, including but not limit to HL-60, Molt-4, and CA46, by markedly downregulating the drug-resistant genes like MDR1, MRP1, TOPIIβ*,* GSTπ, and BCL-2*,* and dramatically blocking AKT/mTOR pathway [205].

With the vigorous development of nanomedicine, nanoscale drug delivery systems have been emergingly recognized as effective technologies for rational drug design and delivery to improve efficacy or diminish systemic adverse effects [206]. Recent progress in the research of emodin-conjugated nanoparticles, for example, based on poly(lactide-co-glycolide)-d-a-tocopheryl polyethylene glycol 1000 succinate, showed impressive biocompatibility, biodegradability, sustained release properties, and highlighted potency as promising therapeutic agents for the treatment of cancers [207, 208]. Besides, emodin-loaded poly (DL-lactide-co-glycolide)/Eudragit^Ⓡ^ S100/montmorillonite nanoparticles specifically released emodin in the diseased colon, thus effectively suppressed ulcerative colitis-related immune-inflammatory responses [209]. Gelatin-cyclodextrin, poly(vinyl pyrrolidone), and cellulose acetate were also applied to assembly-load emodin into different anti-bacteria nanoagent to effectively inhibit the growth of *S. suis* and MRSA [210, 211]. In addition, it has been reported that loading emodin on nanosilver, an efficacious antimicrobial agent, significantly protected against cecal ligation and puncture-induced sepsis in vitro and in vivo, by halting gut-microbiota-mediated systemic infection [212]. Moreover, delivery strategies based on liposomes with different tissue/cancer-targeting properties were widely used to improve the therapeutic effects of emodin. Specifically, targeted liposomes licensed selectively accumulation of emodin at breast cancer lesions, by targeting and inhibiting vasculogenic mimicry channel on breast cancer cell surface, thus exhibiting a distinct antitumor activity in vivo [213, 214]. It was also noteworthy that a magnetically guided liposomal emodin composite was synthesized and enhanced the MCF-7 killing efficiency of hydrophobic free emodin. Intriguingly, under the guidance from the external magnetic field, the effective rate of drug accumulation improved dramatically in the tumor region of 4T1 tumor-bearing mice model, suggesting a novel therapeutic strategy by integratively utilizing physical and biomedical approaches [215].

## Discussion

Rhubarb-containing TCM formulas have been widely used clinically in China since ancient times, and emodin is believed to be the major bioactive compound in rhubarb that possesses arrays of pharmacological properties like anti-inflammation, immunomodulatory, anti-fibrosis, and anti-cancer effects. With the development of modern medicine and system biology, a clearer understanding of molecular mechanisms underlying these biological effects offers experimental for the potential effective clinical application of emodin (Figs.1, 2, and 3). However, there still exist challenges and problems to be solved, thus posing obstacles for further discovery of novel emodin-based therapy.

**Fig. 4:**
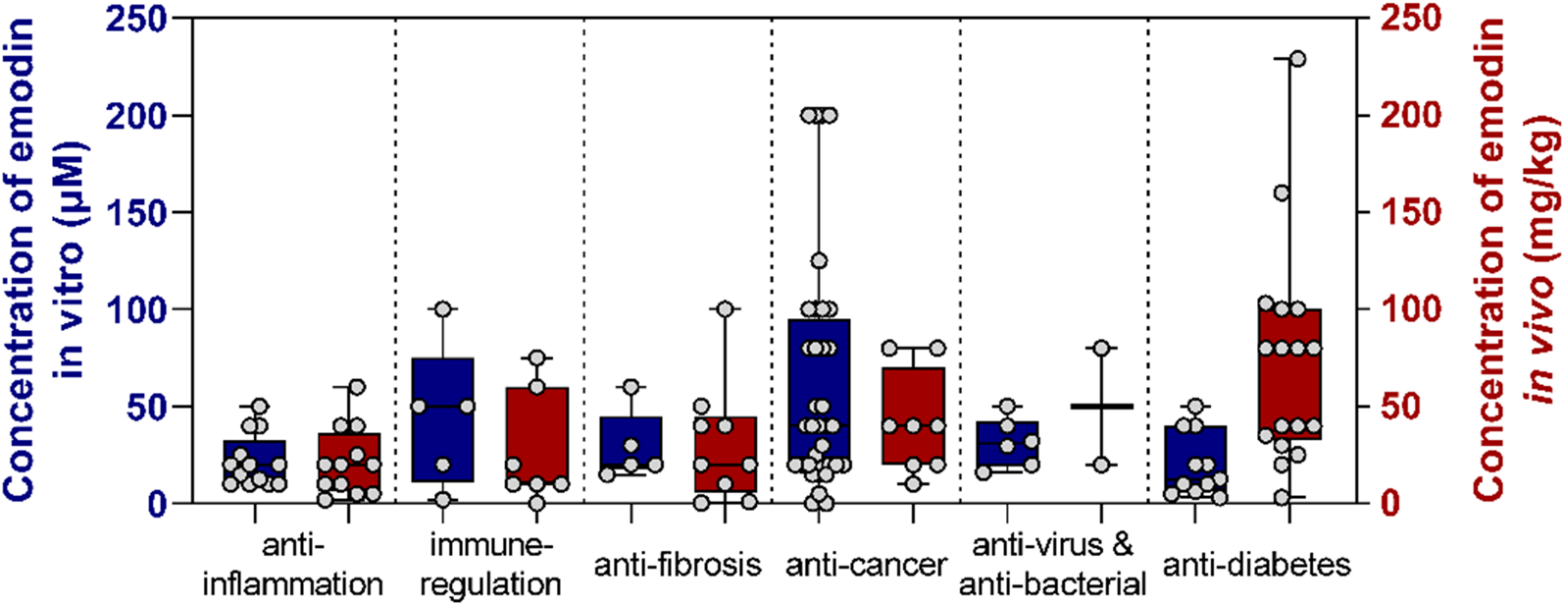
In vivo and in vitro dosage of emodin underlying different pharmacological effects

Primarily, the concentration of emodin used for current in vitro and vivo studies varied considerably and was largely clinically irrelevant. Emodin was reported to exert extensive anticarcinogenic properties, including inhibiting cancer cell proliferation, angiogenesis, metastasis, and inflammation as well as inducing apoptosis, at a wide range of dosage (from 10 to 200 μM), as shown in Fig. 4. What is confusing is that emodin at as low as from 10 to 50 μM displayed other efficacy against inflammatory responses, fibrosis, virus-infection, and diabetes. More intriguingly, no significant differences in oral or intraperitoneal administration dose (ranging from 10 to 80 mg/kg, occasionally at 100 or 150 mg/kg) were observed in rodent models among different diseases. Domestic and foreign researches on systematic pharmacokinetics revealed the low oral and intravenous bioavailability of emodin, illustrated by a long time to reach maximum plasma concentration (T_max_, 2.44 h) and relatively low peak plasma concentration (C_max_, 0.20 μM) of free anthraquinones [216]. Due to the high glucuronidation activity in intestinal and hepatic microsomes, Liu et al*.* detected the relatively high C_max_ of emodin glucuronide (6.69 μg/ml) after single-dose oral 8 mg/kg administration in male rats, supposing the absolute bioavailability of emodin glucuronide was 60% [217]. These results highlighted substantial shortcomings of almost all current in vitro studies, in which an average of more than 10 μM (at least 10 times of C_max_) of emodin in its original form was used to directly treat cells, making these studies less convincing and clinically irrelevant. Additionally, the therapeutic dose window of emodin for the treatment of non-cancerous diseases remains elusive, since the dosages of emodin used for inflammatory, fibrotic, and metabolic diseases were almost the same as the dosage used for cancers. This brought up an issue that emodin might also trigger intolerant adverse effects just like other anti-cancer drugs even it was only used for the intervention of non-malignant disorders. Moreover, the conclusions of several studies are even contradictory, especially when it comes to anti-inflammatory effects and immunoregulatory effects. For instance, the effects of emodin on immune responses, especially on macrophage activation, have drawn great attention over the past decades. Previous studies showed that emodin effectively suppressed excessive macrophage response to M2 stimuli IL-4, via inhibition of IRF4/STAT6 signaling and CCAAT/C/EBPβ signaling. Emodin also epigenetically inhibited changes in H3K27m3 and H3K27ac at the promoter regions of M2 genes to attenuate M2 polarization. However, the promotive effects of emodin on M2 macrophage activation were the primary mechanism underlying its therapeutic effects against asthma. Most recent evidence also reported opposite results that emodin (80 mg/kg) dramatically upregulated trigger receptor expressed on myeloid cells 2 to promote macrophage polarization, thus inhibiting metabolic diseases like obesity and in HFD-mice model [218]. Further studies are required to address these critical issues.

Emodin is considered as the principal antimicrobial component in rhubarb, which exhibits remarkable bacteriostatic or bactericidal effects against Gram-positive organisms, especially *S. aureus*. Historically, rhubarb is a commonly used stimulant laxative with strong purgative potential, drawing increasing attention of translational medicine researchers for its effects on intestinal flora and micro-ecological balance reestablishment in recent years. This laxative effect is mainly attributed to sennoside A, a dianthrone O-glycoside rarely absorbed in small intestine, which can be easily hydrolyzed by intestinal microflora to release rheinanthrone and cause a laxative effect by decreasing aquaporin-3 expression and inhibiting water transport out of colon luminal [219]. Consistently, hydrolysis products aloe-emodin anthrone, rhein anthrone, and emodin were also reported to have synergistic purgative properties through multiple mechanisms, including inhibiting Na^+^/K^+^-ATPase to increase intestinal osmotic pressure, and displaying acetylcholine-like effects to stimulate intestinal motility and peristalsis. These biotransformations among anthraquinones and anthrones by intestinal flora made up for the deficiencies of emodin absorption in small intestinal effectively [191]. A previous study demonstrated that emodin via colonic irrigation altered the gut microbiota structure to improve chronic kidney disease, mainly by reducing uremic toxins-related *Clostridium spp*. and augmenting beneficial probiotic organisms like *Lactobacillus spp*. [220]. Additionally, emodin-contained *Kui-Jie-Yuan* decoction was also reported to improve intestinal flora disturbance and intestinal barrier injury in mice model of ulcerative colitis, including increasing the proportion of *Alloprevotella*, *Treponema*, *Prevotellaceae*, and *Prevotella*, while inhibiting *Escherichia_Shigella* and *Desulfovibrio* [221]. These studies strongly suggested the direct modulatory effects of emodin on not only pathogenic bacteria but also gut-microflora and thus contributed to its pharmacological effects. Emerging evidence suggests that the gut microbiome plays a significant role in modifying tumor immunity as well as modulating the host response to chemotherapy and immunotherapy, so as to influence the outcome of cancers. Most recently, the newly developed cancer fecal microbiota therapy in the clinical trial revealed that fecal microbiota transplant effectively promoted intra-tumoral immune activity and overcame resistance to anti-programmed cell death-1 immunotherapy [222]. Therefore, it is plausible to assume that the potential for regulating gut flora of emodin may help to exert anti-tumor efficiency by affecting immune responses. However, systemic investigation and critical experimental evidence supporting the regulative effects of emodin on gut-microflora homeostasis are still missing.

## Conclusion

In conclusion, although the pharmacological studies at present exist deficiencies as discussed above, emodin still has great potential to become promising therapeutic options to immune and inflammation abnormality, organ fibrosis, common malignancy, pathogenic bacteria or virus infections, and endocrine disease or disorder. Considering the aforementioned issues, further pharmacokinetic study of emodin administration, more accurate dosage design for different diseases, as well as novel drug delivery systems for enhanced bioavailability are with high priorities and urgently required. Scientifically addressing these concerns would significantly contribute to the widespread acceptance of rhubarb not only as a dietary supplement in food flavorings and colorings but also as a health-promoting TCM in the coming years.

## Data Availability

Not applicable.

## Abbreviations

AMPK: AMP-activated protein kinase
AR: Aldose reductase
BCL-2: B-cell lymphoma 2
CVB3: Coxsackievirus B3m
ECM: Extracellular matrix
EGFR: Epidermal growth-factor receptor
EMT: Epithelial-mesenchymal transition
ER: Endoplasmic reticulum
HCC: Hepatocellular carcinoma
HIF-1: Hypoxia-inducible factor-1
IL: Interleukin
ILK: Integrin-linked kinase
lncRNAs: Long-noncoding RNAs
LPS: Lipopolysaccharide
miR: MicroRNAs
MIC: Minimal inhibitory concentration
MMPs: Matrix metalloproteinases
MRSA: Methicillin-resistant *S. aureus*
mTOR: Mammalian target of rapamycin
NF-κB: Nuclear factor kappa-B
NLRP3: NOD-like receptors family pyrin domain containing 3
Nrf2: Nuclear factor erythrocyte-2 associated factor-2
SAP: Severe acute pancreatitis
SMADs: Small mothers against decapentaplegic
SREBPs: Sterol regulatory element-binding proteins
STAT: Signal transducer and activator of transcription
TCM: Traditional Chinese Medicine
TGF-β1: Transforming growth factor beta 1
TLR: Toll-like receptor
TNF-α: Tumor necrosis factor-alpha
TRAF6: TNF receptor-associated factor 6
TUG1: Taurine raised gene 1
VEGF: Vascular endothelial growth factor

## References

[1] Taylor M (2015). Complementary and alternative approaches to menopause. Endocrinol Metab Clin North Am.

[2] Dietz BM, Hajirahimkhan A, Dunlap TL, Bolton JL (2016). Botanicals and their bioactive phytochemicals for women's health. Pharmacol Rev.

[3] Cirillo C, Capasso R (2015). Constipation and botanical medicines: an overview. Phytother Res PTR.

[4] Neyrinck AM, Etxeberria U, Taminiau B, Daube G, Van Hul M, Everard A (2017). Rhubarb extract prevents hepatic inflammation induced by acute alcohol intake, an effect related to the modulation of the gut microbiota. Mol Nutr Food Res..

[5] Lin TJ, Lin CF, Chiu CH, Lee MC, Horng JT (2016). Inhibition of endosomal fusion activity of influenza virus by *Rheum tanguticum* (da-huang). Sci Rep.

[6] Yan J, Xie G, Liang C, Hu Y, Zhao A, Huang F (2017). Herbal medicine Yinchenhaotang protects against alpha-naphthylisothiocyanate-induced cholestasis in rats. Sci Rep.

[7] Hu X, Liu S, Zhu J, Ni H (2019). Dachengqi decoction alleviates acute lung injury and inhibits inflammatory cytokines production through TLR4/NF-kappaB signaling pathway in vivo and in vitro. J Cell Biochem.

[8] Gong Z, Lin J, Zheng J, Wei L, Liu L, Peng Y (2020). Dahuang Zhechong pill attenuates CCl4-induced rat liver fibrosis via the PI3K-Akt signaling pathway. J Cell Biochem.

[9] Huang Q, Lu G, Shen HM, Chung MC, Ong CN (2007). Anti-cancer properties of anthraquinones from rhubarb. Med Res Rev.

[10] Sun J, Wu Y, Dong S, Li X, Gao W (2018). Influence of the drying method on the bioactive compounds and pharmacological activities of rhubarb. J Sci Food Agric.

[11] Li L, Song X, Yin Z, Jia R, Li Z, Zhou X (2016). The antibacterial activity and action mechanism of emodin from *Polygonum cuspidatum* against *Haemophilus parasuis* in vitro. Microbiol Res.

[12] Ahn SM, Kim HN, Kim YR, Choi YW, Kim CM, Shin HK (2016). Emodin from *Polygonum multiflorum* ameliorates oxidative toxicity in HT22 cells and deficits in photothrombotic ischemia. J Ethnopharmacol.

[13] Yang J, Zhu A, Xiao S, Zhang T, Wang L, Wang Q (2019). Anthraquinones in the aqueous extract of *Cassiae semen* cause liver injury in rats through lipid metabolism disorder. Phytomedicine..

[14] Moreira TF, Sorbo JM, Souza FO, Fernandes BC, Ocampos FMM, de Oliveira DMS (2018). Emodin, physcion, and crude extract of *Rhamnus sphaerosperma* var. pubescens induce mixed cell death, increase in oxidative stress, DNA damage, and inhibition of AKT in cervical and oral squamous carcinoma cell lines. Oxid Med Cell Longev..

[15] Dong X, Fu J, Yin X, Cao S, Li X, Lin L (2016). Emodin: a review of its pharmacology, toxicity and pharmacokinetics. Phytotherapy research : PTR.

[16] Song YD, Li XZ, Wu YX, Shen Y, Liu FF, Gao PP (2018). Emodin alleviates alternatively activated macrophage and asthmatic airway inflammation in a murine asthma model. Acta Pharmacol Sin.

[17] Ding Y, Xu J, Cheng LB, Huang YQ, Wang YQ, Li H (2020). Effect of emodin on coxsackievirus B3m mediated-encephalitis in hand, foot and mouth disease by inhibiting toll-like receptor 3 pathway in vitro and in vivo. J Infect Dis..

[18] Wu CC, Chen MS, Cheng YJ, Ko YC, Lin SF, Chiu IM (2019). Emodin inhibits EBV reactivation and represses NPC tumorigenesis. Cancers..

[19] Chang KC, Li L, Sanborn TM, Shieh B, Lenhart P, Ammar D (2016). Characterization of emodin as a therapeutic agent for diabetic cataract. J Nat Prod.

[20] Lin L, Liu Y, Fu S, Qu C, Li H, Ni J (2019). Inhibition of mitochondrial complex function-the hepatotoxicity mechanism of emodin based on quantitative proteomic analyses. Cells..

[21] Wang C, Dai X, Liu H, Yi H, Zhou D, Liu C (2015). Involvement of PPARγ in emodin-induced HK-2 cell apoptosis. Toxicol In Vitro.

[22] Rossi M, Wen K, Caruso F, Belli S (2020). Emodin scavenging of superoxide radical includes π-π Interaction. X-ray crystal structure, hydrodynamic voltammetry and theoretical studies. Antioxidants..

[23] Hoesel B, Schmid JA (2013). The complexity of NF-kappaB signaling in inflammation and cancer. Mol Cancer.

[24] Xu K, Zhou T, Huang Y, Chi Q, Shi J, Zhu P (2018). Anthraquinone emodin inhibits tumor necrosis factor alpha-induced calcification of human aortic valve interstitial cells via the NF-κB pathway. Front Pharmacol.

[25] Luo S, Deng X, Liu Q, Pan Z, Zhao Z, Zhou L (2018). Emodin ameliorates ulcerative colitis by the flagellin-TLR5 dependent pathway in mice. Int Immunopharmacol.

[26] Ding Y, Liu P, Chen ZL, Zhang SJ, Wang YQ, Cai X (2018). Emodin attenuates lipopolysaccharide-induced acute liver injury via inhibiting the TLR4 signaling pathway in vitro and in vivo. Front Pharmacol.

[27] Zhu T, Zhang W, Feng SJ, Yu HP (2016). Emodin suppresses LPS-induced inflammation in RAW264.7 cells through a PPARgamma-dependent pathway. Int Immunopharmacol..

[28] Zhang W, Lu X, Wang W, Ding Z, Fu Y, Zhou X (2017). Inhibitory effects of emodin, thymol, and astragalin on leptospira interrogans-induced inflammatory response in the uterine and endometrium epithelial cells of mice. Inflammation.

[29] Ka SO, Hwang HP, Jang JH, Hyuk Bang I, Bae UJ, Yu HC (2015). The protein kinase 2 inhibitor tetrabromobenzotriazole protects against renal ischemia reperfusion injury. Sci Rep.

[30] Marques-Rocha JL, Samblas M, Milagro FI, Bressan J, Martínez JA, Marti A (2015). Noncoding RNAs, cytokines, and inflammation-related diseases. FASEB J.

[31] Ou C, Li G (2017). Long non-coding RNA TUG1: a novel therapeutic target in small cell lung cancer. J Thorac Dis.

[32] Katsushima K, Natsume A, Ohka F, Shinjo K, Hatanaka A, Ichimura N (2016). Targeting the Notch-regulated non-coding RNA TUG1 for glioma treatment. Nat Commun.

[33] Liang Z, Ren C (2018). Emodin attenuates apoptosis and inflammation induced by LPS through up-regulating lncRNA TUG1 in murine chondrogenic ATDC5 cells. Biomed Pharmacother..

[34] Zang L, Song Y, Yu F, Liu X (2020). Emodin relieved lipopolysaccharide-evoked inflammatory damage in WI-38 cells by up-regulating taurine up-regulated gene 1. BioFactors.

[35] Iorio MV, Croce CM (2009). MicroRNAs in cancer: small molecules with a huge impact. J Clin Oncol.

[36] Dong H, Lei J, Ding L, Wen Y, Ju H, Zhang X (2013). MicroRNA: function, detection, and bioanalysis. Chem Rev.

[37] Shiga A, Nozaki H, Yokoseki A, Nihonmatsu M, Kawata H, Kato T (2011). Cerebral small-vessel disease protein HTRA1 controls the amount of TGF-beta1 via cleavage of proTGF-beta1. Hum Mol Genet.

[38] Xiang H, Tao X, Xia S, Qu J, Song H, Liu J (2017). Emodin Alleviates sodium taurocholate-induced pancreatic acinar cell injury via microRNA-30a-5p-mediated inhibition of high-temperature requirement A/transforming growth factor beta 1 inflammatory signaling. Front Immunol.

[39] Xie R, Liu M, Li S (2019). Emodin weakens liver inflammatory injury triggered by lipopolysaccharide through elevating microRNA-145 in vitro and in vivo. Artif Cells Nanomed Biotechnol.

[40] Jeffries J, Zhou W, Hsu AY, Deng Q (2019). miRNA-223 at the crossroads of inflammation and cancer. Cancer Lett.

[41] Yang Y, Jiang Z, Zhuge D (2019). Emodin attenuates lipopolysaccharide-induced injury via down-regulation of miR-223 in H9c2 cells. Int Heart J.

[42] Song Y, Cui X, Zhao R, Hu L, Li Y, Liu C (2019). Emodin protects against lipopolysaccharide-induced inflammatory injury in HaCaT cells through upregulation of miR-21. Artif Cells Nanomed Biotechnol.

[43] Schroder K, Tschopp J (2010). The inflammasomes. Cell.

[44] Han JW, Shim DW, Shin WY, Heo KH, Kwak SB, Sim EJ (2015). Anti-inflammatory effect of emodin via attenuation of NLRP3 inflammasome activation. Int J Mol Sci.

[45] Giuliani AL, Sarti AC, Falzoni S, Di Virgilio F (2017). The P2X7 receptor-interleukin-1 liaison. Front Pharmacol.

[46] Zhang Q, Tao X, Xia S, Qu J, Song H, Liu J (2019). Emodin attenuated severe acute pancreatitis via the P2X ligandgated ion channel 7/NODlike receptor protein 3 signaling pathway. Oncol Rep.

[47] Zhang Q, Hu F, Guo F, Zhou Q, Xiang H, Shang D (2019). Emodin attenuates adenosine triphosphate-induced pancreatic ductal cell injury in vitro via the inhibition of the P2X7/NLRP3 signaling pathway. Oncol Rep..

[48] Gao Z, Sui J, Fan R, Qu W, Dong X, Sun D (2020). Emodin protects against acute pancreatitis-associated lung injury by inhibiting NLPR3 inflammasome activation via Nrf2/HO-1 signaling. Drug Des Dev Ther.

[49] Xu Q, Wang M, Guo H, Liu H, Zhang G, Xu C (2021). Emodin alleviates severe acute pancreatitis-associated acute lung injury by inhibiting the cold-inducible RNA-binding protein (CIRP)-mediated activation of the NLRP3/IL-1β/CXCL1 signaling. Front Pharmacol..

[50] Ye B, Chen X, Dai S, Han J, Liang X, Lin S (2019). Emodin alleviates myocardial ischemia/reperfusion injury by inhibiting gasdermin D-mediated pyroptosis in cardiomyocytes. Drug Des Dev Ther.

[51] Chen YK, Xu YK, Zhang H, Yin JT, Fan X, Liu DD (2016). Emodin alleviates jejunum injury in rats with sepsis by inhibiting inflammation response. Biomed Pharmacother.

[52] Medford AR, Millar AB (2006). Vascular endothelial growth factor (VEGF) in acute lung injury (ALI) and acute respiratory distress syndrome (ARDS): paradox or paradigm?. Thorax.

[53] Hoeben A, Landuyt B, Highley MS, Wildiers H, Van Oosterom AT, De Bruijn EA (2004). Vascular endothelial growth factor and angiogenesis. Pharmacol Rev.

[54] Li X, Shan C, Wu Z, Yu H, Yang A, Tan B (2020). Emodin alleviated pulmonary inflammation in rats with LPS-induced acute lung injury through inhibiting the mTOR/HIF-1alpha/VEGF signaling pathway. Inflamm Res.

[55] Wamil M, Battle JH, Turban S, Kipari T, Seguret D, de Sousa PR (2011). Novel fat depot-specific mechanisms underlie resistance to visceral obesity and inflammation in 11 beta-hydroxysteroid dehydrogenase type 1-deficient mice. Diabetes.

[56] Esteves CL, Kelly V, Breton A, Taylor AI, West CC, Donadeu FX (2014). Proinflammatory cytokine induction of 11beta-hydroxysteroid dehydrogenase type 1 (11beta-HSD1) in human adipocytes is mediated by MEK, C/EBPbeta, and NF-kappaB/RelA. J Clin Endocrinol Metab.

[57] Lai W, Tian X, Xiang Q, Chu K, Wei Y, Deng J (2015). 11beta-HSD1 modulates LPS-induced innate immune responses in adipocytes by altering expression of PTEN. Mol Endocrinol.

[58] Murray PJ, Wynn TA (2011). Protective and pathogenic functions of macrophage subsets. Nat Rev Immunol.

[59] Iwanowycz S, Wang J, Altomare D, Hui Y, Fan D (2016). Emodin bidirectionally modulates macrophage polarization and epigenetically regulates macrophage memory. J Biol Chem.

[60] Lloyd CM, Hessel EM (2010). Functions of T cells in asthma: more than just T(H)2 cells. Nat Rev Immunol.

[61] Wang T, Zhong XG, Li YH, Jia X, Zhang SJ, Gao YS (2015). Protective effect of emodin against airway inflammation in the ovalbumin-induced mouse model. Chin J Integr Med.

[62] Golubovskaya V, Wu L (2016). Different subsets of T cells, memory, effector functions, and CAR-T immunotherapy. Cancers..

[63] Sharma R, Tiku AB (2016). Emodin inhibits splenocyte proliferation and inflammation by modulating cytokine responses in a mouse model system. J Immunotoxicol.

[64] Pyzik A, Grywalska E, Matyjaszek-Matuszek B, Rolinski J (2015). Immune disorders in Hashimoto's thyroiditis: what do we know so far?. J Immunol Res..

[65] Sun H, Ye Z, Li N, Jin F, Yan J, Wu K (2018). Effect of emodin on T cell subsets in NOD mice with NaIinduced experimental autoimmune thyroiditis. Mol Med Rep.

[66] Qiu F, Liu H, Liang CL, Nie GD, Dai Z (2017). A New Immunosuppressive molecule emodin Induces both CD4(+)FoxP3(+) and CD8(+)CD122(+) regulatory T cells and suppresses murine allograft rejection. Front Immunol.

[67] Shang Y, Smith S, Hu X (2016). Role of Notch signaling in regulating innate immunity and inflammation in health and disease. Protein Cell.

[68] Hua S, Liu F, Wang M (2019). Emodin alleviates the airway inflammation of cough variant asthma in mice by regulating the notch pathway. Med Sci Monit.

[69] Papayannopoulos V (2018). Neutrophil extracellular traps in immunity and disease. Nat Rev Immunol.

[70] Zhu M, Yuan K, Lu Q, Zhu Q, Zhang S, Li X (2019). Emodin ameliorates rheumatoid arthritis by promoting neutrophil apoptosis and inhibiting neutrophil extracellular trap formation. Mol Immunol.

[71] Wang GJ, Wang Y, Teng YS, Sun FL, Xiang H, Liu JJ (2016). Protective effects of emodin-induced neutrophil apoptosis via the Ca(2+)-caspase 12 pathway against SIRS in rats with severe acute pancreatitis. Biomed Res Int.

[72] Ning JW, Zhang Y, Yu MS, Gu ML, Xu J, Usman A (2017). Emodin alleviates intestinal mucosal injury in rats with severe acute pancreatitis via the caspase-1 inhibition. Hepatobiliary Pancreat Dis Inte.

[73] Rockey DC, Bell PD, Hill JA (2015). Fibrosis–a common pathway to organ injury and failure. N Engl J Med.

[74] Meng XM, Nikolic-Paterson DJ, Lan HY (2016). TGF-beta: the master regulator of fibrosis. Nat Rev Nephrol.

[75] Liu F, Zhang J, Qian J, Wu G, Ma Z (2018). Emodin alleviates CCl4induced liver fibrosis by suppressing epithelialmesenchymal transition and transforming growth factorbeta1 in rats. Mol Med Rep.

[76] Zhao XA, Chen G, Liu Y, Wu H, Chen J, Xiong Y (2018). Emodin alleviates liver fibrosis of mice by reducing infiltration of Gr1(hi) monocytes. Evid Based Complement Altern Med.

[77] Wang X, Niu C, Zhang X, Dong M (2018). Emodin suppresses activation of hepatic stellate cells through p38 mitogen-activated protein kinase and Smad signaling pathways in vitro. Phytother Res.

[78] Guan R, Wang X, Zhao X, Song N, Zhu J, Wang J (2016). Emodin ameliorates bleomycin-induced pulmonary fibrosis in rats by suppressing epithelial-mesenchymal transition and fibroblast activation. Sci Rep.

[79] Yang T, Wang J, Pang Y, Dang X, Ren H, Liu Y (2016). Emodin suppresses silica-induced lung fibrosis by promoting Sirt1 signaling via direct contact. Mol Med Rep.

[80] Gao R, Chen R, Cao Y, Wang Y, Song K, Zhang Y (2017). Emodin suppresses TGF-beta1-induced epithelial-mesenchymal transition in alveolar epithelial cells through Notch signaling pathway. Toxicol Appl Pharmacol.

[81] Zhou L, Gao R, Hong H, Li X, Yang J, Shen W (2020). Emodin inhibiting neutrophil elastase-induced epithelial-mesenchymal transition through Notch1 signalling in alveolar epithelial cells. J Cell Mol Med.

[82] Ma L, Li H, Zhang S, Xiong X, Chen K, Jiang P (2018). Emodin ameliorates renal fibrosis in rats via TGF-beta1/Smad signaling pathway and function study of Smurf 2. Int Urol Nephrol.

[83] Pechkovsky DV, Prele CM, Wong J, Hogaboam CM, McAnulty RJ, Laurent GJ (2012). STAT3-mediated signaling dysregulates lung fibroblast-myofibroblast activation and differentiation in UIP/IPF. Am J Pathol.

[84] Guan R, Zhao X, Wang X, Song N, Guo Y, Yan X (2016). Emodin alleviates bleomycin-induced pulmonary fibrosis in rats. Toxicol Lett.

[85] Verfaillie T, Rubio N, Garg AD, Bultynck G, Rizzuto R, Decuypere JP (2012). PERK is required at the ER-mitochondrial contact sites to convey apoptosis after ROS-based ER stress. Cell Death Differ.

[86] Xiong G, Chen H, Wan Q, Dai J, Sun Y, Wang J (2019). Emodin promotes fibroblast apoptosis and prevents epidural fibrosis through PERK pathway in rats. J Orthop Surg Res.

[87] Qin W, Du N, Zhang L, Wu X, Hu Y, Li X (2015). Genistein alleviates pressure overload-induced cardiac dysfunction and interstitial fibrosis in mice. Br J Pharmacol.

[88] Carver W, Fix E, Fix C, Fan D, Chakrabarti M, Azhar M (2021). Effects of emodin, a plant-derived anthraquinone, on TGF-β1-induced cardiac fibroblast activation and function. J Cell Physiol..

[89] Xiao D, Zhang Y, Wang R, Fu Y, Zhou T, Diao H (2019). Emodin alleviates cardiac fibrosis by suppressing activation of cardiac fibroblasts via upregulating metastasis associated protein 3. Acta pharmaceutica Sinica B.

[90] Pistritto G, Trisciuoglio D, Ceci C, Garufi A, D'Orazi G (2016). Apoptosis as anticancer mechanism: function and dysfunction of its modulators and targeted therapeutic strategies. Aging.

[91] Fang N, Zhang W, Xu S, Lin H, Wang Z, Liu H (2014). TRIB3 alters endoplasmic reticulum stress-induced beta-cell apoptosis via the NF-kappaB pathway. Metab Clin Exp..

[92] Su J, Yan Y, Qu J, Xue X, Liu Z, Cai H (2017). Emodin induces apoptosis of lung cancer cells through ER stress and the TRIB3/NF-kappaB pathway. Oncol Rep.

[93] Cui Y, Lu P, Song G, Liu Q, Zhu D, Liu X (2016). Involvement of PI3K/Akt, ERK and p38 signaling pathways in emodin-mediated extrinsic and intrinsic human hepatoblastoma cell apoptosis. Food Chem Toxicol.

[94] Lin W, Zhong M, Yin H, Chen Y, Cao Q, Wang C (2016). Emodin induces hepatocellular carcinoma cell apoptosis through MAPK and PI3K/AKT signaling pathways in vitro and in vivo. Oncol Rep.

[95] Zhang L, He D, Li K, Liu H, Wang B, Zheng L (2017). Emodin targets mitochondrial cyclophilin D to induce apoptosis in HepG2 cells. Biomed Pharmacother..

[96] Ashkenazi A, Fairbrother WJ, Leverson JD, Souers AJ (2017). From basic apoptosis discoveries to advanced selective BCL-2 family inhibitors. Nat Rev Drug Discovery.

[97] Saunders IT, Mir H, Kapur N, Singh S (2019). Emodin inhibits colon cancer by altering BCL-2 family proteins and cell survival pathways. Cancer Cell Int.

[98] Levy JMM, Towers CG, Thorburn A (2017). Targeting autophagy in cancer. Nat Rev Cancer.

[99] Wang Y, Luo Q, He X, Wei H, Wang T, Shao J (2018). Emodin induces apoptosis of colon cancer cells via induction of autophagy in a ROS-dependent manner. Oncol Res.

[100] Trybus W, Krol T, Trybus E, Kopacz-Bednarska A, Krol G, Karpowicz E (2017). Changes in the lysosomal system of cervical cancer cells induced by emodin action. Anticancer Res.

[101] Zhou J, Li G, Han G, Feng S, Liu Y, Chen J (2020). Emodin induced necroptosis in the glioma cell line U251 via the TNF-α/RIP1/RIP3 pathway. Invest New Drugs.

[102] Janssen A, Medema RH (2011). Mitosis as an anti-cancer target. Oncogene.

[103] Recasens A, Munoz L (2019). Targeting cancer cell dormancy. Trends Pharmacol Sci.

[104] Wang Y, Yu H, Zhang J, Ge X, Gao J, Zhang Y (2015). Anti-tumor effect of emodin on gynecological cancer cells. Cell Oncol.

[105] Trybus W, Krol T, Trybus E, Stachurska A, Krol G, Kopacz-Bednarska A (2019). Emodin induces death in human cervical cancer cells through mitotic catastrophe. Anticancer Res.

[106] Deng G, Ju X, Meng Q, Yu ZJ, Ma LB (2015). Emodin inhibits the proliferation of PC3 prostate cancer cells in vitro via the Notch signaling pathway. Mol Med Rep.

[107] Haque E, Kamil M, Irfan S, Sheikh S, Hasan A, Nazir A (2018). Blocking mutation independent p53 aggregation by emodin modulates autophagic cell death pathway in lung cancer. Int J Biochem Cell Biol.

[108] Chen Y, Mei X, Gan D, Wu Z, Cao Y, Lin M (2018). Integration of bioinformatics and experiments to identify TP53 as a potential target in Emodin inhibiting diffuse large B cell lymphoma. Biomed Pharmacother..

[109] Navarro F, Lieberman J (2015). miR-34 and p53: new Insights into a complex functional relationship. PloS ONE..

[110] Bai J, Wu J, Tang R, Sun C, Ji J, Yin Z (2020). Emodin, a natural anthraquinone, suppresses liver cancer in vitro and in vivo by regulating VEGFR(2) and miR-34a. Invest New Drugs.

[111] Dai G, Ding K, Cao Q, Xu T, He F, Liu S (2019). Emodin suppresses growth and invasion of colorectal cancer cells by inhibiting VEGFR2. Eur J Pharmacol..

[112] Masoud GN, Li W (2015). HIF-1α pathway: role, regulation and intervention for cancer therapy. Acta pharmaceutica Sinica B.

[113] Ma F, Hu L, Yu M, Wang F (2016). Emodin decreases hepatic hypoxia-inducible factor-1[Formula: see text] by inhibiting its biosynthesis. Am J Chin Med.

[114] Zheng CC, Hu HF, Hong P, Zhang QH, Xu WW, He QY (2019). Significance of integrin-linked kinase (ILK) in tumorigenesis and its potential implication as a biomarker and therapeutic target for human cancer. Am J Cancer Res.

[115] Tang Q, Zhao S, Wu J, Zheng F, Yang L, Hu J (2015). Inhibition of integrin-linked kinase expression by emodin through crosstalk of AMPKα and ERK1/2 signaling and reciprocal interplay of Sp1 and c-Jun. Cell Signal.

[116] Zhang H, Chen L, Bu HQ, Yu QJ, Jiang DD, Pan FP (2015). Effects of emodin on the demethylation of tumor-suppressor genes in pancreatic cancer PANC-1 cells. Oncol Rep.

[117] Zhou RS, Wang XW, Sun QF, Ye ZJ, Liu JW, Zhou DH (2019). Anticancer effects of emodin on HepG2 cell: evidence from bioinformatic analysis. Biomed Res Int.

[118] Pastushenko I, Blanpain C (2019). EMT transition states during tumor progression and metastasis. Trends Cell Biol.

[119] Li N, Wang C, Zhang P, You S (2018). Emodin inhibits pancreatic cancer EMT and invasion by upregulating microRNA1271. Mol Med Rep.

[120] Lin SZ, Xu JB, Ji X, Chen H, Xu HT, Hu P (2015). Emodin inhibits angiogenesis in pancreatic cancer by regulating the transforming growth factor-β/drosophila mothers against decapentaplegic pathway and angiogenesis-associated microRNAs. Mol Med Rep.

[121] Song K, Lv T, Chen Y, Diao Y, Yao Q, Wang Y (2018). Emodin inhibits TGF-β2 by activating the FOXD3/miR-199a axis in ovarian cancer cells in vitro. Oncol Rep.

[122] Hsu HC, Liu LC, Wang HY, Hung CM, Lin YC, Ho CT (2017). Stromal fibroblasts from the interface zone of triple negative breast carcinomas induced epithelial-mesenchymal transition and its inhibition by emodin. PloS ONE..

[123] Song X, Zhou X, Qin Y, Yang J, Wang Y, Sun Z (2018). Emodin inhibits epithelialmesenchymal transition and metastasis of triple negative breast cancer via antagonism of CCchemokine ligand 5 secreted from adipocytes. Int J Mol Med.

[124] Li Q, Lai Q, He C, Fang Y, Yan Q, Zhang Y (2019). RUNX1 promotes tumour metastasis by activating the Wnt/β-catenin signalling pathway and EMT in colorectal cancer. J Exp Clin Cancer Res.

[125] Liang Z, Lu L, Mao J, Li X, Qian H, Xu W (2017). Curcumin reversed chronic tobacco smoke exposure induced urocystic EMT and acquisition of cancer stem cells properties via Wnt/β-catenin. Cell Death Dis..

[126] Gu J, Cui CF, Yang L, Wang L, Jiang XH (2019). Emodin Inhibits colon cancer cell invasion and migration by suppressing epithelial-mesenchymal transition via the Wnt/beta-catenin pathway. Oncol Res.

[127] Hu C, Dong T, Li R, Lu J, Wei X, Liu P (2016). Emodin inhibits epithelial to mesenchymal transition in epithelial ovarian cancer cells by regulation of GSK-3beta/beta-catenin/ZEB1 signaling pathway. Oncol Rep.

[128] Lu J, Xu Y, Zhao Z, Ke X, Wei X, Kang J (2017). Emodin suppresses proliferation, migration and invasion in ovarian cancer cells by down regulating ILK in vitro and in vivo. Onco Targets Ther.

[129] Lu J, Xu Y, Wei X, Zhao Z, Xue J, Liu P (2016). Emodin inhibits the epithelial to mesenchymal transition of epithelial ovarian cancer cells via ILK/GSK-3beta/Slug signaling pathway. Biomed Res Int.

[130] Sun H, Li XB, Meng Y, Fan L, Li M, Fang J (2013). TRAF6 upregulates expression of HIF-1alpha and promotes tumor angiogenesis. Can Res.

[131] Gabison EE, Hoang-Xuan T, Mauviel A, Menashi S (2005). EMMPRIN/CD147, an MMP modulator in cancer, development and tissue repair. Biochimie.

[132] Shi GH, Zhou L (2018). Emodin suppresses angiogenesis and metastasis in anaplastic thyroid cancer by affecting TRAF6-mediated pathways in vivo and in vitro. Mol Med Rep.

[133] Lin W, Zhong M, Liang S, Chen Y, Liu D, Yin Z (2016). Emodin inhibits migration and invasion of MHCC-97H human hepatocellular carcinoma cells. Exp Ther Med.

[134] Kim JM, Noh EM, Song HK, You YO, Jung SH, Kim JS (2018). Silencing of casein kinase 2 inhibits PKC-induced cell invasion by targeting MMP-9 in MCF-7 cells. Mol Med Rep.

[135] Su S, Liu Q, Chen J, Chen J, Chen F, He C (2014). A positive feedback loop between mesenchymal-like cancer cells and macrophages is essential to breast cancer metastasis. Cancer Cell.

[136] Iwanowycz S, Wang J, Hodge J, Wang Y, Yu F, Fan D (2016). Emodin inhibits breast cancer growth by blocking the tumor-promoting feedforward loop between cancer cells and macrophages. Mol Cancer Ther.

[137] Liu Q, Hodge J, Wang J, Wang Y, Wang L, Singh U (2020). Emodin reduces breast cancer lung metastasis by suppressing macrophage-induced breast cancer cell epithelial-mesenchymal transition and cancer stem cell formation. Theranostics.

[138] Gao J, Zheng Q, Xin N, Wang W, Zhao C (2017). CD155, an onco-immunologic molecule in human tumors. Cancer Sci.

[139] Fang L, Zhao F, Iwanowycz S, Wang J, Yin S, Wang Y (2019). Anticancer activity of emodin is associated with downregulation of CD155. Int Immunopharmacol..

[140] Fridlender ZG, Sun J, Kim S, Kapoor V, Cheng G, Ling L (2009). Polarization of tumor-associated neutrophil phenotype by TGF-beta: "N1" versus "N2" TAN. Cancer Cell.

[141] Li Z, Lin Y, Zhang S, Zhou L, Yan G, Wang Y (2019). Emodin regulates neutrophil phenotypes to prevent hypercoagulation and lung carcinogenesis. J Transl Med.

[142] Keating GM (2017). Sorafenib: a review in hepatocellular carcinoma. Target Oncol.

[143] Kim YS, Lee YM, Oh TI, Shin DH, Kim GH, Kan SY (2018). Emodin sensitizes hepatocellular carcinoma cells to the anti-cancer effect of sorafenib through suppression of cholesterol metabolism. Int J Mol Sci..

[144] Chen S, Zhang Z, Zhang J (2019). Emodin enhances antitumor effect of paclitaxel on human non-small-cell lung cancer cells in vitro and in vivo. Drug Des Dev Ther.

[145] Phng LK, Potente M, Leslie JD, Babbage J, Nyqvist D, Lobov I (2009). Nrarp coordinates endothelial Notch and Wnt signaling to control vessel density in angiogenesis. Dev Cell.

[146] Zu C, Qin G, Yang C, Liu N, He A, Zhang M (2018). Low dose emodin induces tumor senescence for boosting breast cancer chemotherapy via silencing NRARP. Biochem Biophys Res Commun.

[147] Pan FP, Zhou HK, Bu HQ, Chen ZQ, Zhang H, Xu LP (2016). Emodin enhances the demethylation by 5-Aza-CdR of pancreatic cancer cell tumor-suppressor genes P16, RASSF1A and ppENK. Oncol Rep.

[148] Thomas NA, Abraham RG, Dedi B, Krucher NA (2019). Targeting retinoblastoma protein phosphorylation in combination with EGFR inhibition in pancreatic cancer cells. Int J Oncol.

[149] Wang Z, Chen H, Chen J, Hong Z, Liao Y, Zhang Q (2019). Emodin sensitizes human pancreatic cancer cells to EGFR inhibitor through suppressing Stat3 signaling pathway. Cancer Manag Res.

[150] Hintzpeter J, Seliger JM, Hofman J, Martin HJ, Wsol V, Maser E (2016). Inhibition of human anthracycline reductases by emodin—a possible remedy for anthracycline resistance. Toxicol Appl Pharmacol.

[151] Li X, Wang H, Wang J, Chen Y, Yin X, Shi G (2016). Emodin enhances cisplatin-induced cytotoxicity in human bladder cancer cells through ROS elevation and MRP1 downregulation. BMC Cancer.

[152] Iyer VV, Priya PY, Kangeyavelu J (2018). Effects of increased accumulation of doxorubicin due to emodin on efflux transporter and LRP1 expression in lung adenocarcinoma and colorectal carcinoma cells. Mol Cell Biochem.

[153] Batista MN, Braga ACS, Campos GRF, Souza MM, Matos RPA, Lopes TZ (2019). Natural products isolated from oriental medicinal herbs inactivate Zika virus. Viruses..

[154] Dai JP, Wang QW, Su Y, Gu LM, Zhao Y, Chen XX (2017). Emodin inhibition of influenza A virus replication and influenza viral pneumonia via the Nrf2, TLR4, p38/JNK and NF-kappaB pathways. Molecules.

[155] Zhong T, Zhang LY, Wang ZY, Wang Y, Song FM, Zhang YH (2017). Rheum emodin inhibits enterovirus 71 viral replication and affects the host cell cycle environment. Acta Pharmacol Sin.

[156] Zhang HM, Wang F, Qiu Y, Ye X, Hanson P, Shen H (2016). Emodin inhibits coxsackievirus B3 replication via multiple signalling cascades leading to suppression of translation. Biochem J.

[157] Hall CW, Mah TF (2017). Molecular mechanisms of biofilm-based antibiotic resistance and tolerance in pathogenic bacteria. FEMS Microbiol Rev.

[158] Yang YB, Wang S, Wang C, Huang QY, Bai JW, Chen JQ (2015). Emodin affects biofilm formation and expression of virulence factors in *Streptococcus suis* ATCC700794. Arch Microbiol.

[159] Yan X, Gu S, Shi Y, Cui X, Wen S, Ge J (2017). The effect of emodin on *Staphylococcus aureus* strains in planktonic form and biofilm formation in vitro. Arch Microbiol.

[160] Liu M, Peng W, Qin R, Yan Z, Cen Y, Zheng X (2015). The direct anti-MRSA effect of emodin via damaging cell membrane. Appl Microbiol Biotechnol.

[161] Ji X, Liu X, Peng Y, Zhan R, Xu H, Ge X (2017). Comparative analysis of methicillin-sensitive and resistant *Staphylococcus aureus* exposed to emodin based on proteomic profiling. Biochem Biophys Res Commun.

[162] Li L, Tian Y, Yu J, Song X, Jia R, Cui Q (2017). iTRAQ-based quantitative proteomic analysis reveals multiple effects of emodin to *Haemophilus parasuis*. J Proteomics.

[163] Harikrishnan R, Jawahar S, Thamizharasan S, Paray BA, Al-Sadoon MK, Balasundaram C (2018). Immune defense of emodin enriched diet in *Clarias batrachus* against *Aeromonas hydrophila*. Fish Shellfish Immunol.

[164] Jung HA, Ali MY, Choi JS (2016). Promising inhibitory effects of anthraquinones, naphthopyrone, and naphthalene glycosides, from *Cassia obtusifolia* on α-glucosidase and human protein tyrosine phosphatases 1B. Molecules..

[165] Arvindekar A, More T, Payghan PV, Laddha K, Ghoshal N, Arvindekar A (2015). Evaluation of anti-diabetic and alpha glucosidase inhibitory action of anthraquinones from *Rheum emodi*. Food Funct.

[166] Lamers D, Famulla S, Wronkowitz N, Hartwig S, Lehr S, Ouwens DM (2011). Dipeptidyl peptidase 4 is a novel adipokine potentially linking obesity to the metabolic syndrome. Diabetes.

[167] Wang Z, Yang L, Fan H, Wu P, Zhang F, Zhang C (2017). Screening of a natural compound library identifies emodin, a natural compound from *Rheum palmatum* Linn that inhibits DPP4. PeerJ..

[168] Yu L, Gong L, Wang C, Hu N, Tang Y, Zheng L (2020). Radix polygoni multiflori and its main component emodin attenuate non-alcoholic fatty liver disease in zebrafish by regulation of AMPK signaling pathway. Drug Des Dev Ther.

[169] Zhang X, Zhang R, Lv P, Yang J, Deng Y, Xu J (2015). Emodin up-regulates glucose metabolism, decreases lipolysis, and attenuates inflammation in vitro. J Diabetes.

[170] Wang S, Li X, Guo H, Yuan Z, Wang T, Zhang L (2017). Emodin alleviates hepatic steatosis by inhibiting sterol regulatory element binding protein 1 activity by way of the calcium/calmodulin-dependent kinase kinase-AMP-activated protein kinase-mechanistic target of rapamycin-p70 ribosomal S6 kinase signaling pathway. Hepatol Res.

[171] Li X, Xu Z, Wang S, Guo H, Dong S, Wang T (2016). Emodin ameliorates hepatic steatosis through endoplasmic reticulum-stress sterol regulatory element-binding protein 1c pathway in liquid fructose-feeding rats. Hepatol Res.

[172] Shimano H, Sato R (2017). SREBP-regulated lipid metabolism: convergent physiology—divergent pathophysiology. Nat Rev Endocrinol.

[173] Li J, Ding L, Song B, Xiao X, Qi M, Yang Q (2016). Emodin improves lipid and glucose metabolism in high fat diet-induced obese mice through regulating SREBP pathway. Eur J Pharmacol.

[174] Xiao D, Hu Y, Fu Y, Wang R, Zhang H, Li M (2019). Emodin improves glucose metabolism by targeting microRNA-20b in insulin-resistant skeletal muscle. Phytomedicine.

[175] Cao Y, Chang S, Dong J, Zhu S, Zheng X, Li J (2016). Emodin ameliorates high-fat-diet induced insulin resistance in rats by reducing lipid accumulation in skeletal muscle. Eur J Pharmacol.

[176] Cheng L, Zhang S, Shang F, Ning Y, Huang Z, He R (2021). Emodin improves glucose and lipid metabolism disorders in obese mice via activating brown adipose tissue and inducing browning of white adipose tissue. Front Endocrinol..

[177] Abu Eid S, Adams M, Scherer T, Torres-Gómez H, Hackl MT, Kaplanian M (2017). Emodin, a compound with putative antidiabetic potential, deteriorates glucose tolerance in rodents. Eur J Pharmacol.

[178] Quattrini L, La Motta C (2019). Aldose reductase inhibitors: 2013-present. Expert Opin Ther Pat.

[179] Tian N, Gao Y, Wang X, Wu X, Zou D, Zhu Z (2018). Emodin mitigates podocytes apoptosis induced by endoplasmic reticulum stress through the inhibition of the PERK pathway in diabetic nephropathy. Drug Des Dev Ther.

[180] Jing D, Bai H, Yin S (2017). Renoprotective effects of emodin against diabetic nephropathy in rat models are mediated via PI3K/Akt/GSK-3β and Bax/caspase-3 signaling pathways. Exp Ther Med.

[181] Xu S, Lv Y, Zhao J, Wang J, Zhao X, Wang S (2016). Inhibitory effects of Shenkang injection and its main component emodin on the proliferation of high glucose-induced renal mesangial cells through cell cycle regulation and induction of apoptosis. Mol Med Rep.

[182] Schalkwijk CG, Stehouwer CDA (2020). Methylglyoxal, a highly reactive dicarbonyl compound, in diabetes, its vascular complications, and other age-related diseases. Physiol Rev.

[183] Sohn E, Kim J, Kim CS, Jo K, Kim JS (2015). Extract of Rhizoma *Polygonum cuspidatum* reduces early renal podocyte injury in streptozotocin-induced diabetic rats and its active compound emodin inhibits methylglyoxal-mediated glycation of proteins. Mol Med Rep.

[184] Fan L, Zhang H, Li X, Yang G, Ru J, Liu T (2018). Emodin protects hyperglycemia-induced injury in PC-12 cells by up-regulation of miR-9. Mol Cell Endocrinol.

[185] Zhang Y, Yang X, Jia Z, Liu J, Yan X, Dai Y (2020). Proteomics unravels emodin causes liver oxidative damage elicited by mitochondrial dysfunction. Front Pharmacol.

[186] Chen C, Gao J, Wang TS, Guo C, Yan YJ, Mao CY (2018). NMR-based metabolomic techniques identify the toxicity of emodin in HepG2 cells. Sci Rep.

[187] Luo T, Li N, He YQ, Weng SQ, Wang T, Zou QX (2015). Emodin inhibits human sperm functions by reducing sperm [Ca(2+)]i and tyrosine phosphorylation. Reprod Toxicol.

[188] Teng Z, Yuan C, Zhang F, Huan M, Cao W, Li K (2012). Intestinal absorption and first-pass metabolism of polyphenol compounds in rat and their transport dynamics in Caco-2 cells. PloS ONE..

[189] Qin B, Xu Y, Chen J, Huang W, Peng Y, Zheng J (2016). Chemical reactivity of emodin and its oxidative metabolites to thiols. Chem Res Toxicol.

[190] Xu Y, Wang Q, Yin Z, Gao X (2018). On-line incubation and real-time detection by ultra-performance liquid chromatography-quadrupole time-of-flight mass spectrometry for rapidly analyzing metabolites of anthraquinones in rat liver microsomes. J Chromatogr A.

[191] Huang Z, Xu Y, Wang Q, Gao X (2019). Metabolism and mutual biotransformations of anthraquinones and anthrones in rhubarb by human intestinal flora using UPLC-Q-TOF/MS. J Chromatogr B.

[192] Park B, Yoon W, Yun J, Ban E, Yun H, Kim A (2019). Emodin-nicotinamide (1:2) cocrystal identified by thermal screening to improve emodin solubility. Int J Pharm.

[193] Liu W, Tang L, Ye L, Cai Z, Xia B, Zhang J (2010). Species and gender differences affect the metabolism of emodin via glucuronidation. AAPS J.

[194] Ban E, Park M, Jeong S, Kwon T, Kim EH, Jung K (2017). Poloxamer-based thermoreversible gel for topical delivery of emodin: influence of P407 and P188 on solubility of emodin and its application in cellular activity screening. Molecules..

[195] Campos PP, Fraceto LF, Ferreira M (2018). Layer-by-layer films containing emodin or emodin encapsulated in liposomes for transdermal applications. Colloids Surf B.

[196] Wei W, Meng C, Wang Y, Huang Y, Du W, Li H (2019). The interaction between self—assembling peptides and emodin and the controlled release of emodin from in-situ hydrogel. Artif Cells Nanomed Biotechnol.

[197] Huang J, Gong W, Chen Z, Huang J, Chen Q, Huang H (2017). Emodin self-emulsifying platform ameliorates the expression of FN, ICAM-1 and TGF-β1 in AGEs-induced glomerular mesangial cells by promoting absorption. Eur J Pharm Sci.

[198] Liu W, Feng Q, Li Y, Ye L, Hu M, Liu Z (2012). Coupling of UDP-glucuronosyltransferases and multidrug resistance-associated proteins is responsible for the intestinal disposition and poor bioavailability of emodin. Toxicol Appl Pharmacol.

[199] Wu W, Hu N, Zhang Q, Li Y, Li P, Yan R (2014). In vitro glucuronidation of five rhubarb anthraquinones by intestinal and liver microsomes from humans and rats. Chem Biol Interact.

[200] Zhang T, Dong D, Lu D, Wang S, Wu B (2016). Cremophor EL-based nanoemulsion enhances transcellular permeation of emodin through glucuronidation reduction in UGT1A1-overexpressing MDCKII cells. Int J Pharm.

[201] Akkol EK, Tatlı II, Karatoprak G, Ağar OT, Yücel Ç, Sobarzo-Sánchez E (2021). Is emodin with anticancer effects completely innocent? Two sides of the coin. Cancers..

[202] Yang K, Jin MJ, Quan ZS, Piao HR (2019). Design and synthesis of novel anti-proliferative emodin derivatives and studies on their cell cycle arrest, apoptosis pathway and migration. Molecules.

[203] Khan H, Jia W, Yu Z, Zaib T, Feng J, Jiang Y (2020). Emodin succinyl ester inhibits malignant proliferation and migration of hepatocellular carcinoma by suppressing the interaction of AR and EZH2. Biomed Pharmacother..

[204] Koerner SK, Hanai JI, Bai S, Jernigan FE, Oki M, Komaba C (2017). Design and synthesis of emodin derivatives as novel inhibitors of ATP-citrate lyase. Eur J Med Chem.

[205] Chen Y, Zheng J, Gan D, Chen Y, Zhang N, Chen Y (2020). E35 ablates acute leukemia stem and progenitor cells in vitro and in vivo. J Cell Physiol.

[206] Malam Y, Loizidou M, Seifalian AM (2009). Liposomes and nanoparticles: nanosized vehicles for drug delivery in cancer. Trends Pharmacol Sci.

[207] Liu H, Gao M, Xu H, Guan X, Lv L, Deng S (2016). A Promising emodin-loaded poly (lactic-co-glycolic Acid)-d-α-tocopheryl polyethylene glycol 1000 succinate nanoparticles for liver cancer therapy. Pharm Res.

[208] Dong H, Wu G, Xu H, Zhang C, Wang J, Gao M (2018). N-acetylaminogalactosyl-decorated biodegradable PLGA-TPGS copolymer nanoparticles containing emodin for the active targeting therapy of liver cancer. Artif Cells Nanomed Biotechnol.

[209] Wang D, Sun M, Zhang Y, Chen Z, Zang S, Li G (2020). Enhanced therapeutic efficacy of a novel colon-specific nanosystem loading emodin on DSS-induced experimental colitis. Phytomedicine..

[210] Ding W, Sun J, Lian H, Xu C, Liu X, Zheng S (2018). The Influence of shuttle-shape emodin nanoparticles on the *Streptococcus suis* Biofilm. Front Pharmacol.

[211] Ye P, Wei S, Luo C, Wang Q, Li A, Wei F (2020). Long-term Effect against methicillin-resistant *Staphylococcus aureus* of emodin released from coaxial electrospinning nanofiber membranes with a biphasic profile. Biomolecules..

[212] Li H, Yang T, Zhou H, Du J, Zhu B, Sun Z (2016). Emodin combined with nanosilver inhibited sepsis by anti-inflammatory protection. Front Pharmacol.

[213] Fu M, Tang W, Liu JJ, Gong XQ, Kong L, Yao XM (2020). Combination of targeted daunorubicin liposomes and targeted emodin liposomes for treatment of invasive breast cancer. J Drug Target.

[214] Liu H, Zhuang Y, Wang P, Zou T, Lan M, Li L (2021). Polymeric lipid hybrid nanoparticles as a delivery system enhance the antitumor effect of emodin in vitro and in vivo. J Pharm Sci..

[215] Song Y, Sheng Z, Xu Y, Dong L, Xu W, Li F (2019). Magnetic liposomal emodin composite with enhanced killing efficiency against breast cancer. Biomater Sci.

[216] Wu W, Yan R, Yao M, Zhan Y, Wang Y (2014). Pharmacokinetics of anthraquinones in rat plasma after oral administration of a rhubarb extract. Biomed Chromatogr.

[217] Liu W, Zheng Z, Liu X, Gao S, Ye L, Yang Z (2011). Sensitive and robust UPLC-MS/MS method to determine the gender-dependent pharmacokinetics in rats of emodin and its glucuronide. J Pharm Biomed Anal.

[218] Yu F, Yu N, Peng J, Zhao Y, Zhang L, Wang X (2021). Emodin inhibits lipid accumulation and inflammation in adipose tissue of high-fat diet-fed mice by inducing M2 polarization of adipose tissue macrophages. FASEB J..

[219] Kon R, Ikarashi N, Nagoya C, Takayama T, Kusunoki Y, Ishii M (2014). Rheinanthrone, a metabolite of sennoside A, triggers macrophage activation to decrease aquaporin-3 expression in the colon, causing the laxative effect of rhubarb extract. J Ethnopharmacol.

[220] Zeng YQ, Dai Z, Lu F, Lu Z, Liu X, Chen C (2016). Emodin via colonic irrigation modulates gut microbiota and reduces uremic toxins in rats with chronic kidney disease. Oncotarget.

[221] Liu B, Piao X, Niu W, Zhang Q, Ma C, Wu T (2020). Kuijieyuan decoction improved intestinal barrier injury of ulcerative colitis by affecting TLR4-dependent PI3K/AKT/NF-κB oxidative and inflammatory signaling and gut microbiota. Front Pharmacol.

[222] Baruch EN, Youngster I, Ben-Betzalel G, Ortenberg R, Lahat A, Katz L (2021). Fecal microbiota transplant promotes response in immunotherapy-refractory melanoma patients. Science.

